# Decreased *GZMB*, *NRP1*, *ITPR1*, and *SERPINB9* Transcripts Lead to Reduced Regulatory T Cells Suppressive Capacity in Generalized Vitiligo Patients

**DOI:** 10.1155/2022/3426717

**Published:** 2022-09-15

**Authors:** Prashant S. Giri, Ankit H. Bharti, Mitesh Dwivedi

**Affiliations:** ^1^C. G. Bhakta Institute of Biotechnology, Faculty of Science, Uka Tarsadia University, Bardoli, Surat, 394 350 Gujarat, India; ^2^Independent Researcher, Vyara, Gujarat, India

## Abstract

Generalized vitiligo (GV) is an autoimmune skin disease characterized by bilateral white patches over the entire body. Regulatory T cells (Tregs) maintain peripheral tolerance; however, they are found to be reduced in numbers and function in vitiligo patients. The exact mechanism for reduced Treg suppressive capacity is unknown. Therefore, we aimed to assess transcript levels of Tregs-associated immunosuppressive genes (*GZMB*, *NRP1*, *PDCD1*, *FASLG*, and *TNFRS18*), regulatory molecules of Tregs suppressive function (*SERPINB9*, *ITPR1*, and *UBASH3A*), and Treg-associated transcription factors (*GATA2*, *GATA3*, *RUNX1*, *STAT3*, and *STAT5*) in 52 GV patients and 48 controls by real-time PCR (qPCR). We found significantly reduced *GZMB*, *NRP1*, *SERPINB9*, and *ITPR1* transcripts in GV Tregs compared to controls (*p* = 0.03, *p* = 0.023, *p* = 0.0045, and *p* < 0.0001, respectively). There were 0.44-, 0.45-, 0.32-, and 0.54-fold decrease in *GZMB*, *NRP1*, *SERPINB9*, and *ITPR1* transcripts in GV Tregs. Additionally, disease activity and severity-based analyses revealed significantly decreased *GZMB* (*p* = 0.019 and 0.034), *SERPINB9* (*p* = 0.031 and *p* = 0.035), and *ITPR1* (*p* = 0.0003 and *p* = 0.034) transcripts in active vitiligo and severe GV patients' Tregs. Interestingly, we found a positive correlation for *ITPR1* with *GZMB* (*r* = 0.45, *p* = 0.0009) and *SERPINB9* (*r* = 0.52, *p* = 0.001) transcripts in GV Tregs. Moreover, we found positive correlation for percentage Treg mediated suppression of CD4^+^ and CD8^+^T cells with *ITPR1* (*r* = 0.54; *r* = 0.49), *GZMB* (*r* = 0.61; *r* = 0.58), *NRP1* (*r* = 0.55; *r* = 0.52), and *SERPINB9* (*r* = 0.56; *r* = 0.48) in GV Tregs. Further, calcium treatment of Tregs resulted into significantly increased *ITPR1*, *SERPINB9*, and *GZMB* transcripts in GV Tregs (*p* = 0.023, *p* = 0.0345, *p* = 0.02). Overall, our results for the first time revealed the crucial role of *GZMB*, *NRP1*, *SERPINB9*, and *ITPR1* transcripts in decreased Treg suppressive capacity leading to GV pathogenesis, progression, and severity. In addition, our study highlighted that *ITPR1* might be linked with decreased *GZMB* and *NRP1* expression in GV Tregs. Moreover, our study for the first time suggest that increased *SERPINB9* transcripts may lead to endogenous granzyme B-mediated Tregs apoptosis, and calcium treatment of Tregs may improve the Treg suppressive capacity. These findings may further aid in development of Treg-based therapeutics for GV.

## 1. Introduction

Generalized vitiligo (GV) is an autoimmune disease characterized by symmetrical white patches on the entire body [1]. Its prevalence is about 0.5 to 2% worldwide [2]. The key role of autoimmunity in GV has been suggested by the presence of autoantibodies and autoreactive CD4^+^ and CD8^+^ T cells in vitiligo skin lesions [3]. Furthermore, if they remain unchecked, these autoreactive T cells can lead to granzyme and FAS-FASL-mediated melanocyte destruction, leading to GV pathogenesis [4–8]. Additionally, our previous studies have found the role of inflammatory cytokines IFN-*γ* and TNF-*α* in melanocyte destruction [6, 9–11].

Regulatory T cells (Tregs) are crucial role in controlling such self-reactive T cells [12, 13]. However, previous studies have found altered Tregs number and function in vitiligo [6, 14, 15]. Moreover, our previous studies suggested impaired levels of the transcription factors of Tregs, such as nuclear factors of activated T cells (NFATs) and Forkhead box P3 (FOXP3) that led to reduced downstream immunosuppressive genes (IL-10, TGF-*β*, and CTLA-4), resulting in impaired Treg-mediated suppression of CD4^+^ and CD8^+^ T cells [6, 15, 16]. Thus, the impaired Treg suppressive capacity leads to widespread CD8^+^ and CD4^+^ T cells activation, proliferation, and IFN-*γ* production, which results in melanocyte destruction in GV patients [6, 15, 16].

In Tregs, the key immunosuppressive molecules such as granzyme B (*GZMB*), neuropilin-1 (*NRP1*), PDCD1 (programmed cell death protein 1 (PD-1) or CD279), FASLG (Fas ligand (FasL) or CD95L or CD178), and TNFRS18 (glucocorticoid-induced TNFR-related protein (GITR) or CD357) maintain Treg suppressive function [17–20]. Additionally, SERPINB9 (Serpin family B member 9) endogenous inhibitor of granzyme B protects Tregs from self-inflicted granzyme B-mediated apoptosis [21]. Moreover, inositol 1,4,5-trisphosphate receptor type 1 (ITPR1) regulates calcium entry in T cells. Furthermore, UBASH3A (ubiquitin associated and SH3 domain-containing A) governs T cells' function by regulating the TCR-CD3 complex [22]. Nevertheless, transcription factors such as GATA-binding factor 2 (GATA2), GATA3, runt-related transcription factor 1 (RUNX1), signal transducer and activator of transcription 3 (STAT3), and STAT5 also play a critical role in Treg cells' function [23–27]. Although the role of NFATs and FOXP3 in Treg dysfunction has been suggested previously, the role of Tregs-associated immunosuppressive genes (*GZMB*, *NRP1*, *PDCD1*, *FASLG*, and *TNFRS18*), regulatory molecules of Tregs function (*SERPINB9*, *ITPR1*, and *UBASH3A*), and Treg-associated transcription factors (*GATA2*, *GATA3*, *RUNX1*, *STAT3*, and *STAT5*) is unknown in GV.

Therefore, to delineate the exact pathway of Treg cells dysfunction, the current study aimed to study the mRNA expression levels of (i) Tregs-associated immunosuppressive genes (*GZMB*, *NRP1*, *PDCD1*, *FASLG*, and *TNFRS18*), (ii) regulatory molecules of Tregs function (*SERPINB9*, *ITPR1*, and *UBASH3A*), and (iii) Treg associated transcription factors (*GATA2*, *GATA3*, *RUNX1*, *STAT3*, and *STAT5*) in GV pathogenesis, progression, and severity. Additionally, we carried out the age of onset and gender-based analysis for these genes to study their effect on age of onset and gender biasness for GV pathogenesis.

## 2. Materials and Methods

### 2.1. Study Population

A total of 52 GV patients and 48 healthy controls were included in the study.  Table 1 depicts the demographic details for the enrolled participants. Vitiligo was diagnosed by a dermatologist from Aura skin care clinic, Vyara, India, by observing symmetrical white color lesions on skin under woods lamps. Newborn babies, pregnant/lactating women, patients on treatment, and patients with other autoimmune diseases were excluded from the study. Controls were free from any signs of vitiligo and other autoimmune diseases. The study protocol was per the Institutional Human Research Ethics Committee (IHREC), Maliba Pharmacy College, UKA Tarsadia University, India. The study protocol followed the Helsinki Declaration of 1964 and its successful amendments. The GV patients were divided into active vitiligo (AV) patients and stable vitiligo (SV) patients as mentioned previously [15]. The patients were categorized as AV, if they developed any increase in lesions size or number within the past six months; otherwise, they were categorized as SV [28]. Moreover, patients were categorized based on the disease severity measured by the vitiligo area scoring index (VASI) as described by Bhor and Pande [29]. GV patients were divided into three groups: (i) 10%-25% VASI: mild GV; (ii) 25%-50% VASI: moderate GV; and (iii) 50%-75%: severe GV, as mentioned previously [6].

### 2.2. Isolation of CD4^+^CD25^+^ Treg Cells and CD4^+^ T Cells

**Figure 1:**
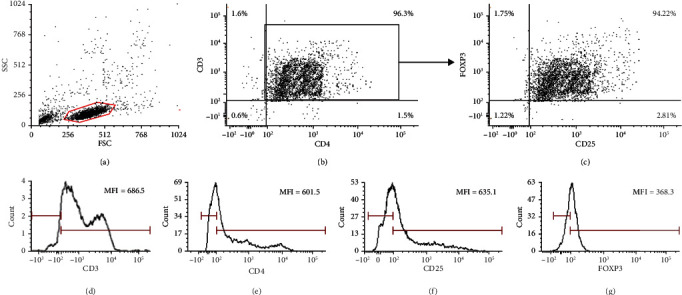
Gating strategy for CD4^+^CD25^+^FOXP3^+^ Treg cells. Estimation of protein levels of CD4, and CD25. (a) Lymphocytes were gated on the basis of size and morphology. (b) CD3^+^CD4^+^T cells were gated on the basis of CD3 and CD4 expression.(c) Treg cells were gated on the basis of CD3, CD4, CD25, and FOXP3 expression. The purity of isolated CD3^+^CD4^+^CD25^+^FOXP3^+^ Treg cells was found to be 94.22%. (d) Expression of CD3 in T cells. Representative graph shows the amount of CD3 in the T cells as mean fluorescence intensity (MFI). (e) Expression of CD4 in T cells. Representative graph shows amount of CD4 in the T cells as mean fluorescence intensity (MFI). (f) Expression of CD25 in T cells. Representative graph shows amount of CD25 in the T cells as mean fluorescence intensity (MFI). (g) Expression of FOXP3 in T cells. Representative graph shows amount of intracellular FOXP3 in the T cells as mean fluorescence intensity (MFI).

CD4^+^CD25^+^ Treg cells and CD4^+^ T cells were isolated from three-milliliter peripheral blood of 52 GV patients and 48 controls using MACSxpress® whole blood Treg isolation kit (Miltenyi Biotec, Auburn, CA) as mentioned previously [15]. In the first step, all the non-CD4^+^ cells were removed from the whole blood using MACSxpress beads by negative selection. In the second step, through positive selection, the CD4^+^CD25^+^ Treg cells were enriched from CD4^+^ T cells using microbeads and LS columns under a strong magnetic field. Flow cytometry was carried out to confirm the purity of isolated Treg cells (Figure 1), the purity of isolated CD3^+^CD4^+^CD25^+^FOXP3^+^ Treg cells was found to be 94.22%. The isolated Treg cells were immediately processed for the downstream experiments.

### 2.3. Isolation of CD8^+^ T Cells

CD8^+^ T cells were isolated from two-milliliter blood sample of GV patients and controls using MACSxpress® whole blood CD8 T cell isolation kit human (Miltenyi Biotec, Auburn, CA) according to the manufacturer's instructions and as mentioned previously [6]. All the non-CD8 T cells were immunomagnetically depleted with MACSxpress beads. The isolated CD8^+^ T cells were immediately processed for *in vitro* Treg suppression assay.

### 2.4. Total RNA Isolation and cDNA Synthesis

The total RNA was extracted from CD4^+^CD25^+^ Treg cells using TRIzol reagent (Invitrogen, Carlsbad, CA, USA) as mentioned previously [15]. RNA integrity, yield, and purity were determined by 1.5% gel electrophoresis and spectrophotometrically at 260/280 nm. The cDNA was synthesized from 1 *μ*g of total RNA by iScript™ cDNA Synthesis Kit (Bio-Rad, CA, USA) as per the manufacturer's instructions.

### 2.5. Quantitative Real-Time PCR

The mRNA expression levels of Treg-associated genes *GZMB*, *NRP1*, *ITPR1*, *SERPINB9*, *PDCD1*, *FASLG*, *UBASH3A*, *IKZF4*, *GATA2*, *GATA3*, *TNFRSF18*, *RUNX1*, *STAT3*, and *STAT5* were measured with qPCR. Glyceraldehyde 3-phosphate dehydrogenase (*GAPDH*) gene expression levels were used as a reference gene. Gene-specific primers for the expression study are mentioned in Table S1. mRNA expression analysis was carried out using iTaq Universal SYBR Green Supermix (Bio-Rad, CA, USA) as per the manufacturer's instructions. The qPCR conditions for the gene expressions study are mentioned in Table S1. The specificity of the qPCR products was checked by dissociation curve analysis (Figures S1 and S2). The fluorescence data were collected during the extension step and the cycle at which the fluorescence intensity raised above the background was termed as cycle threshold (*C*_*T*_). The difference between the target and reference genes *C*_*T*_ was considered as Δ*C*_*T*_ value. The ΔΔ*C*_*T*_ value was determined as the difference between the Δ*C*_*T*_ value of controls and patients. The fold change value in gene expression was calculated using 2^-*ΔΔ*CT^ formula.

### 2.6. *In Vitro* Calcium Treatment of Treg Cells

Treg cells were subjected to calcium treatment by dissolving 750 *μ*M calcium (Cayman, MI, USA) in RPMI media. The dissolved calcium level in the medium was confirmed using calcium assay kit (Cayman, MI, USA). Treg cells were seeded in 24-well plate at density of 5 × 10^4^ cells, in 1 ml RPMI medium supplemented with 5% FBS containing desired calcium concentration at 37°C at 5% CO_2_ for 24 hours. The calcium-treated Treg cells were immediately processed for downstream *in vitro* functional assays. The standard curve for the estimation of calcium is presented in Figure S3.

### 2.7. *In Vitro* Treg Suppression Assay

CD4^+^CD25^+^ Treg cells (5000 cells) were co-cultured with CD8^+^ T cells and CD4^+^ T cells (10,000 cells) at a ratio of 1 : 2 individually, as mentioned previously [6]. The cells were activated with 200 IU rIL2 (PeproTech, NJ, USA) and anti-CD3/CD28 dynabeads Gibco; Thermo Fisher Scientific, Inc., Waltham, MA, USA) at a 1 : 1 (bead:cells) ratio in 200 *μ*l RPMI supplemented with 10% fetal bovine serum for 5 days at 37°C and 5% CO_2_ in 96 well U-bottom plate. On 4th day, the cells were labelled with 10 *μ*M BrdU (Sigma-Aldrich, MO, USA) and incubated for 18 hrs and then further processed for BrdU cell proliferation assay as mentioned previously [6, 30, 31].

### 2.8. BrdU Cell Proliferation Assay

Incorporation of BrdU in proliferating cells was measured by BrdU cell proliferation enzyme-linked immunosorbent assay kit (Sigma-Aldrich, Missouri, USA) according to the manufacturer's instructions. Percentage suppression was calculated using the following formula: [(proliferation of Tconv cells alone–proliferation of Tconv cells treated with Treg)/proliferation of Tconv cells alone] x 100 [6, 31].

### 2.9. Correlation of *ITPR1*, *GZMB*, *NRP1*, and *SERPINB9* Transcripts with *In Vitro* Treg Suppressive Capacity

The isolated Tregs population was divided into two fractions. The inherent levels of *ITPR1*, *GZMB*, *NRP1*, and *SERPINB9* transcripts were assessed from the first fraction, whereas *in vitro* Treg suppression assay was carried out from the second Treg fraction. Further, the inherent levels of *ITPR1*, *GZMB*, *NRP1*, and *SERPINB9* transcripts in Tregs of GV were correlated with the Treg suppressive capacity by Spearman's rank correlation analysis.

### 2.10. Statistical Analysis

The comparison of mean ∆*C*_*T*_ values in GV patients and controls for relative mRNA expression analysis, disease activity analysis, disease severity analysis, age of onset analysis, and gender-based analysis was carried out using nonparametric Mann–Whitney *U* test. The 2^-*ΔΔ*CT^ analysis was carried out to calculate the fold difference in gene expression. The statistical analysis was carried out using GraphPad prism software (Graphpad software Inc.; San Diego, CA, USA, 2003). *p* ≤ 0.05 was considered statistically significant.

## 3. Results

### 3.1. Transcript Levels of Tregs Associated Immunosuppressive Genes (*GZMB*, *NRP1*, *PDCD1*, *FASLG*, and *TNFRS18*) in GV Patients and Controls

**Figure 2:**
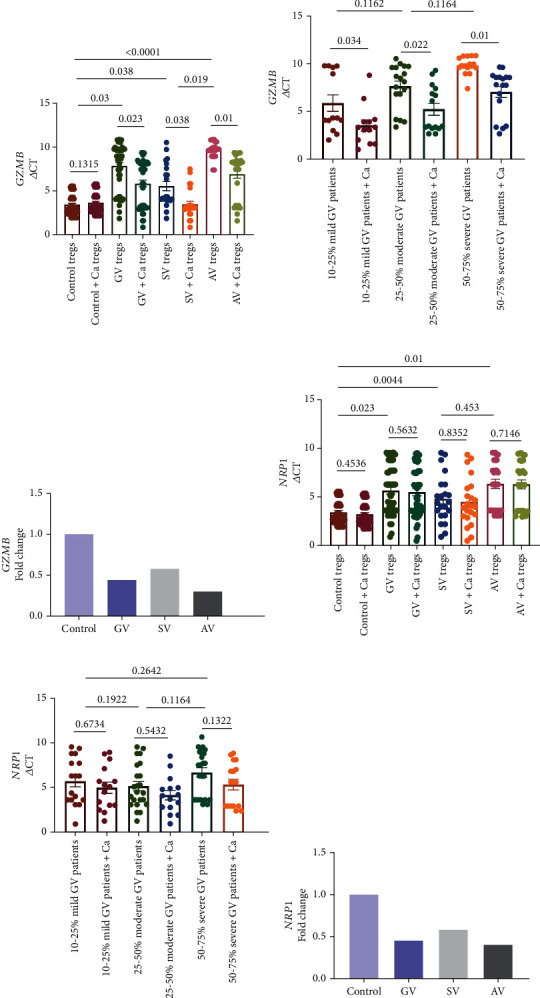
*GZMB* and *NRP1* transcripts in Tregs of GV patients and controls. *GZMB* and *NRP1* transcripts in Tregs of 52 GV patients and 48 controls were analyzed by Nonparametric Mann–Whitney *U* test. (a) *GZMB* transcripts in GV, SV, and AV vs controls' Tregs (*p* = 0.03, *p* = 0.038, and *p* < 0.0001, respectively). *GZMB* transcripts in AV vs SV Tregs (*p* = 0.019). *GZMB* transcripts in GV, SV, and AV Tregs before and after calcium treatment (*p* = 0.023, *p* = 0.0328, and *p* = 0.01). (b) *GZMB* transcripts in 50-75% severe GV vs 10-25% mild GV Tregs (*p* = 0.034). *GZMB* transcripts in moderate GV (25-50% VASI) vs mild GV (10-25% VASI) and severe GV (50-75% VASI) Tregs (*p* = 0.1162 and *p* = 0.1164, respectively). *GZMB* transcripts in mild GV, moderate GV, and severe GV Tregs before and after calcium treatment (*p* = 0.034, *p* = 0.022, and *p* = 0.01). (c) There was a 0.44-, 0.58-, and 0.30-fold changes in *GZMB* transcripts for GV, SV, and AV Tregs as compared to controls. (d) *NRP1* transcripts in GV, SV, and AV vs controls' Tregs (*p* = 0.023, *p* = 0.044, and *p* = 0.001, respectively). *NRP1* transcripts in AV vs SV Tregs (*p* = 0.453). *NRP1* transcripts in GV, SV, and AV Tregs before and after calcium treatment (*p* = 0.5632, *p* = 0.8352, and *p* = 0.7146). (e) *NRP1* transcripts in severe GV (50-75% VASI) vs mild GV (10-25% VASI) Tregs (*p* = 0.2642). *NRP1* transcripts in moderate GV (25-50% VASI) vs mild GV (10-25% VASI) and severe GV (50-75% VASI) Tregs (*p* = 0.1922 and *p* = 0.1164, respectively). *NRP1* transcripts in mild GV, moderate GV, and severe GV Tregs before and after calcium treatment (*p* = 0.6734, *p* = 0.5432, and *p* = 0.1322). (f) There was a 0.45-, 0.58-, and 0.40-fold changes in *NRP1* transcripts for GV, SV, and AV Tregs when compared to controls.

The transcript levels of Tregs-associated immunosuppressive genes (*GZMB*, *NRP1*, *PDCD1*, *FASLG*, and *TNFRS18*) were assessed in 52 GV patients and 48 controls using nonparametric Mann–Whitney *U* test after normalization with *GAPDH* expression. We found significantly reduced transcript levels for *GZMB* and *NRP1* expression in GV Tregs compared to control Tregs (*p* = 0.03 and *p* = 0.023; Figures 2(a) and 2(d)). The 2^-*ΔΔ*CT^ analysis suggested a 0.44- and 0.45-fold difference in mRNA expression levels of *GZMB* and *NRP1* in GV Tregs compared to control Tregs (Figures 2(c) and 2(e)). Further, the disease activity and disease severity-based analysis suggested significantly reduced mRNA expression levels of *GZMB* in AV and severe GV Tregs compared to SV and mild GV Tregs (*p* = 0.019 and *p* = 0.034, respectively; Figures 2(a) and 2(b)). However, there was no significant difference in mRNA expression levels of *NRP1* in AV and severe GV Tregs compared to SV and mild GV Tregs (*p* = 0.453 and *p* = 0.2642; Figures 2(d) and 2(e)). Additionally, we did not find any significant difference in transcripts levels of *PDCD1*, *FASLG*, and *TNFRS18* in GV Tregs compared to control Tregs (*p* > 0.05; Figure S4). Moreover, the disease activity and disease severity-based analysis suggested no significant difference in transcripts levels of *PDCD1*, *FASLG*, and *TNFRS18* in AV and severe GV Tregs compared to SV and mild GV Tregs (*p* > 0.05; Figure S4).

### 3.2. Transcript Levels of *SERPINB9*, *ITPR1*, and *UBASH3A* (Regulatory Molecules of Treg Function) in GV Patients and Controls

**Figure 3:**
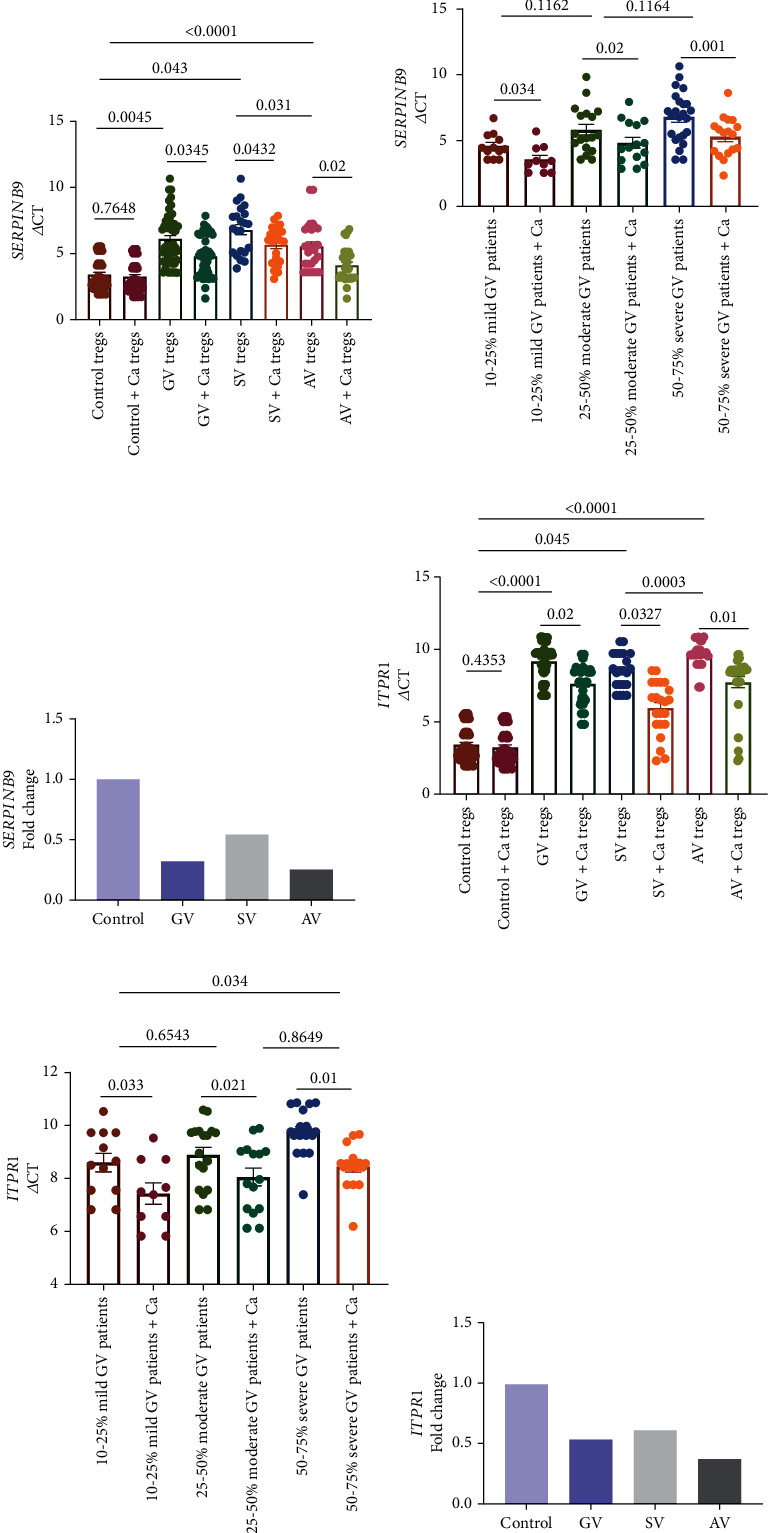
*SERPINB9* and *ITPR1* transcripts in Tregs of GV patients and controls. *SERPINB9* and *ITPR1* transcripts in Tregs of 52 GV patients and 48 controls were analyzed by nonparametric Mann–Whitney *U* test. (a) *SERPINB9* transcripts in GV, SV, and AV vs controls' Tregs (*p* = 0.0045, *p* = 0.043, and *p* < 0.0001, respectively). *SERPINB9* transcripts in AV vs SV Tregs (*p* = 0.031). *SERPINB9* transcripts in GV, SV, and AV Tregs before and after calcium treatment (*p* = 0.0345, *p* = 0.0432, and *p* = 0.02). (b) *SERPINB9* transcripts in severe GV (50-75% VASI) vs mild GV (10-25% VASI) Tregs (*p* = 0.035). *SERPINB9* transcripts in moderate GV (25-50% VASI) vs mild GV (10-25% VASI) and severe GV (50-75% VASI) Tregs (*p* = 0.1162 and *p* = 0.1164, respectively). *SERPINB9* transcripts in mild GV, moderate GV, and severe GV Tregs before and after calcium treatment (*p* = 0.034, *p* = 0.02, and *p* = 0.001, respectively). (c) There was a 0.32-, 0.54-, and 0.25-fold changes in *SERPINB9* transcripts for GV, SV, and AV Tregs when compared to controls. (d) *ITPR1* transcripts in GV, SV, and AV vs controls' Tregs (*p* < 0.0001, *p* = 0.045, and *p* < 0.0001, respectively). *ITPR1* transcripts in AV vs SV Tregs (*p* = 0.0003). *ITPR1* transcripts in GV, SV, and AV Tregs before and after calcium treatment (*p* = 0.02, *p* = 0.0327, and *p* = 0.01). (e) *ITPR1* transcripts in severe GV (50-75% VASI) vs mild GV (10-25% VASI) Tregs (*p* = 0.034). *ITPR1* transcripts in moderate GV (25-50% VASI) vs mild GV (10-25% VASI) and severe GV (50-75% VASI) Tregs (*p* = 0.6543 and *p* = 0.8649, respectively). *ITPR1* transcripts in mild GV, moderate GV, and severe GV Tregs before and after calcium treatment (*p* = 0.033, *p* = 0.021, and *p* = 0.01). (f) There was a 0.54-, 0.62-, and 0.28-fold changes in *ITPR1* transcripts for GV, SV, and AV Tregs when compared to controls.

The transcript levels for regulatory molecules of Treg function (*SERPINB9*, *ITPR1*, and *UBASH3A*) were assessed in 52 GV patients and 48 controls using nonparametric Mann–Whitney *U* test after normalization with *GAPDH* expression. Our study suggested significant decrease in transcript levels of *SERPINB9* and *ITPR1* for GV Tregs compared to controls' Tregs (*p* = 0.045 and *p* < 0.0001; Figures 3(a) and 3(d)). According to the 2^-*ΔΔ*CT^ analysis, there was a 0.32- and 0.54-fold difference in transcript levels of *SERPINB9* and *ITPR1* in GV Tregs compared to controls' Tregs (Figures 3(c) and 3(f)). Moreover, the disease activity and severity-based analysis suggested a significantly decreased mRNA expression levels of *SERPINB9* and *ITPR1* in AV Tregs (*p* = 0.031 and *p* = 0.0003; Figures 3(a) and 3(d)) and severe GV Tregs (*p* = 0.035 and *p* = 0.034; Figures 3(b) and 3(e)) as compared to SV and mild GV Tregs. However, we could not find any significant difference in transcript levels of *UBASH3A* for GV Tregs as compared to controls' Tregs (*p* = 0.145; Figure S5). The disease activity and severity-based analysis also suggested no significant difference in mRNA expression levels of *UBASH3A* in AV and severe GV Tregs compared to SV and mild GV Tregs (*p* = 0.487; Figure S5).

### 3.3. Transcripts Levels of Treg Associated Transcription Factors (*GATA2*, *GATA3*, *RUNX1*, *STAT3*, and *STAT5*) in GV Patients and Controls

The transcript levels for Treg-associated transcription factors (*GATA2*, *GATA3*, *RUNX1*, *STAT3*, and *STAT5*) were assessed in 52 GV patients and 48 controls using nonparametric Mann–Whitney *U* test after normalization with *GAPDH* expression. We did not find any significant difference for transcript levels of *GATA2*, *GATA3*, *RUNX1*, *STAT3*, and *STAT5* in GV Tregs when compared to controls' Tregs (*p* > 0.05; Figures S5 and S6). Moreover, the disease activity and severity-based analysis also suggested no significant difference in mRNA expression levels of *GATA2*, *GATA3*, *RUNX1*, *STAT3*, and *STAT5* in AV and severe GV Tregs as compared to SV and mild GV Tregs (*p* > 0.05; Figures S5 and S6).

### 3.4. Effect of Calcium Treatment on *ITPR1*, *GZMB*, *NRP1*, and *SERPINB9* Transcripts in GV Patients' and Controls' Tregs

Previously, we have found that calcium treatment enhances the calcium uptake in Tregs resulting in increased NFATc1 activity which leads to enhanced Treg suppressive capacity in GV [32]. As *ITPR1*governs the release of calcium in Tregs and *GZMB*, *NRP1*, and *SERPINB9* are crucial for Treg cells activity, we studied the effect of calcium treatment on *ITPR1*, *GZMB*, *NRP1*, and *SERPINB9* transcripts in Tregs of GV patients and controls. Upon calcium treatment, we found significantly increased *ITPR1* transcripts in Tregs of GV, SV, and AV patients compared to controls (*p* = 0.02, *p* = 0.0327, and *p* = 0.01; Figure 3(d)). Interestingly, the calcium treatment led to an increased *SERPINB9* (*p* = 0.0345, *p* = 0.0432, and *p* = 0.02; Figure 3(a)) and *GZMB* (*p* = 0.023, *p* = 0.038, and *p* = 0.01; Figure 2(a)) transcripts in calcium-treated Tregs of GV, SV, and AV patients, respectively, compared to untreated Tregs of GV, SV, and AV patients, respectively. Moreover, the calcium treatment led to increased *ITPR1* (*p* = 0.033, *p* = 0.021, and *p* = 0.01; Figure 3(e)), *GZMB* (*p* = 0.034, *p* = 0.022, and *p* = 0.01; Figure 2(b)), and *SERPINB9* (*p* = 0.034, *p* = 0.02, and *p* = 0.001; Figure 3(b)) transcripts in mild GV, moderate GV and severe GV Tregs. We did not find any significant difference in *NRP1* transcripts in calcium-treated Tregs of GV, SV, AV, mild GV, moderate GV, and severe GV patients (*p* > 0.05; Figures 2(d) and 2(e)).

### 3.5. Correlation of *ITPR1* Transcripts with *GZM*B, *NRP1*, and *SERPINB9* Transcripts in Tregs and Correlation of *In Vitro* Treg Suppression Assay with of *ITPR1*, *GZMB*, *NRP1*, and *SERPINB9* Transcripts in Tregs of GV Patients

**Figure 4:**
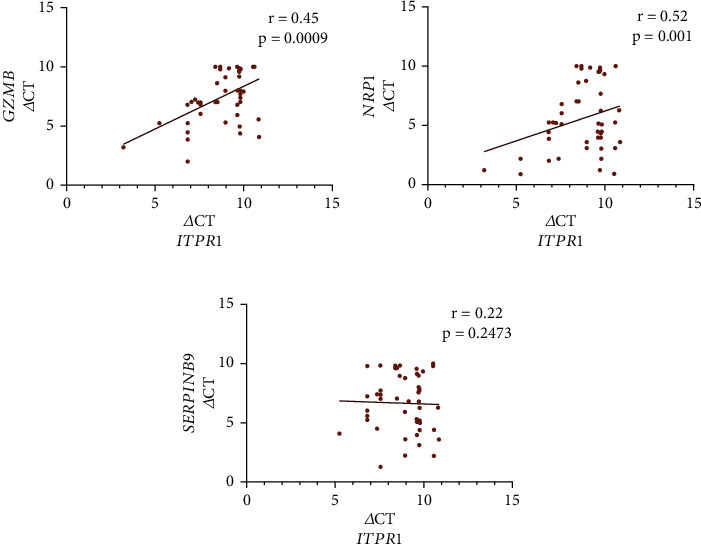
Correlation of *ITPR1* transcripts with *GZMB*, *NRP1*, and *SERPINB9* transcripts in GV patients. The correlation of *ITPR1* transcripts with *GZMB*, *NRP1*, and *SERPINB9* transcripts were analyzed by Spearman's correlation analysis. (a) *ITPR1* transcripts positively correlated with *GZMB* transcripts in GV patients' Tregs (*r* = 0.45, *p* = 0.0009). (b) *ITPR1* transcripts positively correlated with *NRP1* transcripts in GV patients' Tregs (*r* = 0.52, *p* = 0.001). (c) No correlation was observed between *ITPR1* transcripts and *SERPINB9* transcripts in GV patients' Tregs (*r* = 0.22, *p* = 0.2473).

**Figure 5:**
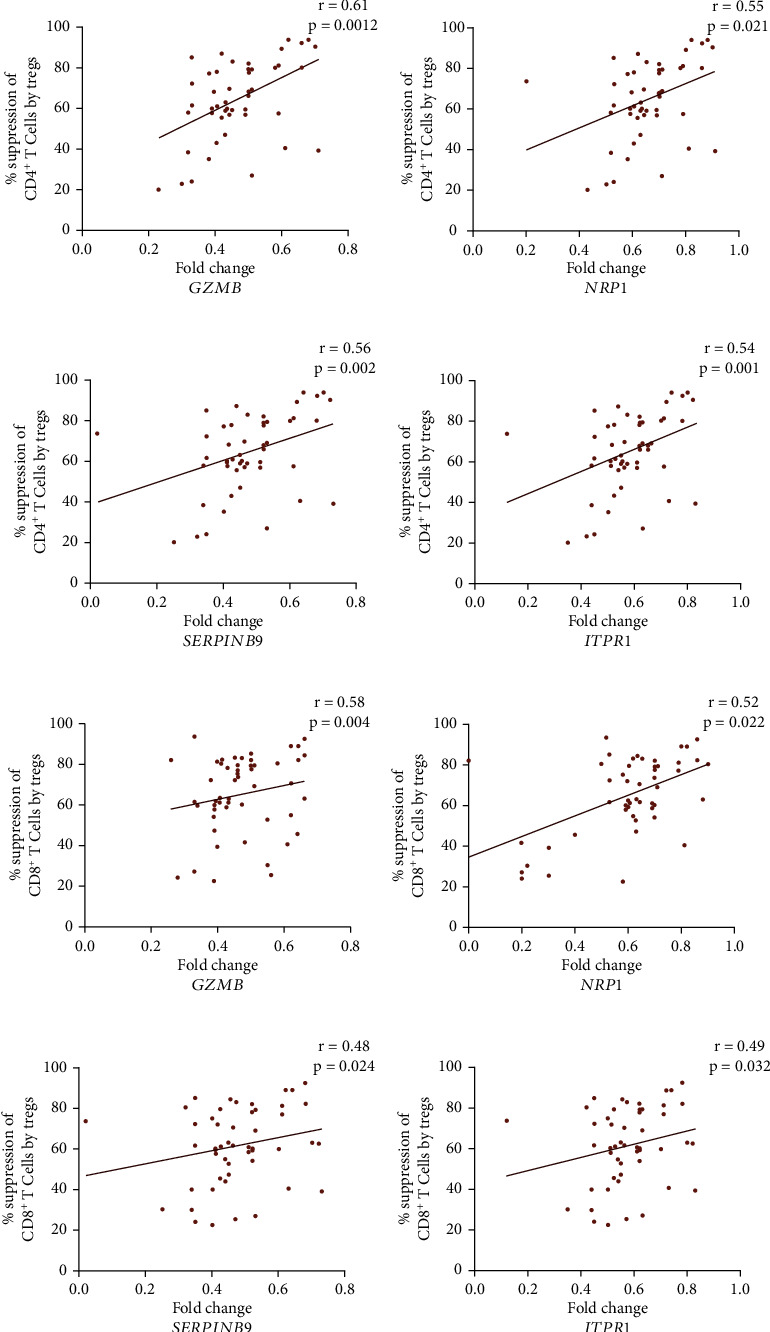
Correlation of *in vitro* Treg suppression assay with *ITPR1*, *GZMB*, *NRP1*, and *SERPINB9* transcripts in Tregs of GV patients. The correlation of *in vitro* Treg suppression assay with *ITPR1*, *GZMB*, *NRP1*, and *SERPINB9* was analyzed by Spearman's correlation analysis. (a) *GZMB* transcripts were positively correlated with *in vitro* Treg mediated suppression of CD4^+^ T cells in GV patients' Tregs (*r* = 0.61; *p* = 0.0012). (b) *NRP1* transcripts were positively correlated with *in vitro* Treg-mediated suppression of CD4^+^ T cells in GV patients' Tregs (*r* = 0.55; *p* = 0.021). (c) *SERPINB9* transcripts were positively correlated with *in vitro* Treg-mediated suppression of CD4^+^ T cells in GV patients' Tregs (*r* = 0.56; *p* = 0.002). (d) *ITPR1* transcripts were positively correlated with *in vitro* Treg-mediated suppression of CD4^+^ T cells in GV patients' Tregs (*r* = 0.54; *p* = 0.001). (e) *GZMB* transcripts positively correlated with *in vitro* Treg-mediated suppression of CD8^+^ T cells in GV patients' Tregs (*r* = 0.58; *p* = 0.004). (f) *NRP1* transcripts positively correlated with *in vitro* Treg-mediated suppression of CD8^+^ T cells in GV patients' Tregs (*r* = 0.52; *p* = 0.0022). (g) *SERPINB9* transcripts positively correlated with *in vitro* Treg-mediated suppression of CD8^+^ T cells in GV patients' Tregs (*r* = 0.48; *p* = 0.024). (h) *ITPR1* transcripts positively correlated with *in vitro* Treg-mediated suppression of CD8^+^ T cells in GV patients' Tregs (*r* = 0.49; *p* = 0.032).

Interestingly, we found a positive correlation for *ITPR1* transcripts with GZMB (*r* = 0.45; *p* = 0.0009) and *NRP1* (*r* = 0.52; *p* = 0.001) transcripts in Tregs of GV patients (Figures 4(a) and 4(b)). However, we could not find any correlation between *ITPR1* and *SERPINB9* transcripts in Tregs of GV patients (*r* = 0.22; *p* = 0.2473; Figure 4(c)). Further, we correlated *in vitro* Treg suppression assay with *GZMB*, *NRP1*, *SERPINB9*, and *ITPR1* transcripts in Tregs of GV patients. We found a positive correlation for percentage Treg-mediated suppression of CD4^+^ T cells with *GZMB* (*r* = 0.61; *p* = 0.0012), *NRP1* (*r* = 0.55; *p* = 0.021), *SERPINB9* (*r* = 0.56; *p* = 0.002), and *ITPR1* (*r* = 0.54; *p* = 0.001) and percentage Treg-mediated suppression of CD8^+^ T cells with *GZMB* (*r* = 0.58; *p* = 0.004), *NRP1* (*r* = 0.52; *p* = 0.022), *SERPINB9* (*r* = 0.48; *p* = 0.024), and *ITPR1* (*r* = 0.49; *p* = 0.032) in GV patients (Figures 5(a)–5(h)).

### 3.6. Age of Onset and Gender-Based Analyses for Transcripts Levels of Treg-Associated Genes (*GZMB*, *NRP1*, *ITPR1*, *SERPINB9*, *PDCD1*, *FASLG*, *UBASH3A*, *IKZF4*, *GATA2*, *GATA3*, *TNFRSF18*, *RUNX1*, *STAT3*, and *STAT5*) in GV Patients

Further, the expression of *GZMB*, *NRP1*, *ITPR1*, *SERPINB9*, *PDCD1*, *FASLG*, *UBASH3A*, *IKZF4*, *GATA2*, *GATA3*, *TNFRSF18*, *RUNX1*, *STAT3*, and *STAT5* transcripts was monitored in different age at onset groups of GV patients. We did not find any significant difference in the expression of *GZMB*, *NRP1*, *ITPR1*, *SERPINB9*, *PDCD1*, *FASLG*, *UBASH3A*, *IKZF4*, *GATA2*, *GATA3*, *TNFRSF18*, *RUNX1*, *STAT3*, and *STAT5* transcripts in Tregs between 1–20, 21-40, and 41-60 years age of onset groups (*p* > 0.05; Figures S7 and S8).

Next, we carried out gender-based analysis for expression of *GZMB*, *NRP1*, *ITPR1*, *SERPINB9*, *PDCD1*, *FASLG*, *UBASH3A*, *IKZF4*, *GATA2*, *GATA3*, *TNFRSF18*, *RUNX1*, *STAT3*, and *STAT5* transcripts. We did not find any significant difference in expression of *GZMB*, *NRP1*, *ITPR1*, *SERPINB9*, *PDCD1*, *FASLG*, *UBASH3A*, *IKZF4*, *GATA2*, *GATA3*, *TNFRSF18*, *RUNX1*, *STAT3*, and *STAT5* transcripts between male and female GV Tregs (*p* > 0.05; Figures S9 and S10).

## 4. Discussion

Autoimmunity has been strongly implicated in GV pathogenesis by the presence of autoantibodies and autoreactive CD4^+^ and CD8^+^ T cells in GV patients [3–8]. Additionally, studies have implicated the role of cytotoxic T cells in melanocyte death in vitiligo patients [7, 8, 15]. Tregs control such autoimmune responses against melanocytes by actively suppressing self-reactive T cells activation and expansion [13, 14]. However, studies have suggested impaired Tregs' number and Tregs suppressive function in GV patients [6, 14, 15]. Moreover, our recent study has suggested the role of altered expression of NFATs and FOXP3 in impaired Treg suppressive function [6]. Thus, impaired Tregs fail to control the ongoing autoimmune response leading to widespread self-reactive T cells activation, resulting in GV pathogenesis [6]. Overall, these studies highlight that Tregs and their suppressive molecules may represent a potential therapeutic target for developing Treg-based therapeutics for GV.

**Figure 6:**
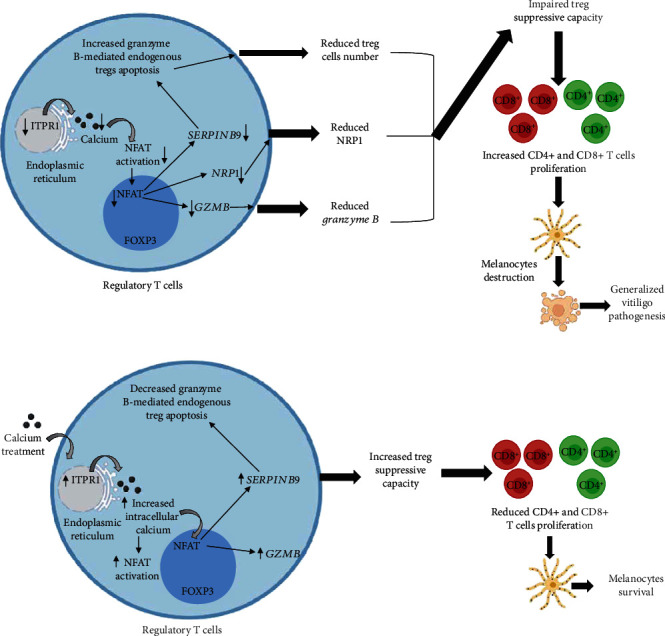
Role of *GZMB*, *NRP1*, *SERPINB9*, and *ITPR1* transcripts in GV pathogenesis. (a) The decreased *ITPR1* transcripts could lead to impaired calcium-NFAT signalling pathway, which might result in decreased *GZMB* and NRP1 transcripts. Further, the decreased *SERPINB9* transcripts may result in increased granzyme B-mediated endogenous apoptosis of Tregs. Overall, the decreased *GZMB*, *NRP1*, *SERPINB9*, and *ITPR1* transcripts result into decreased Treg suppressive capacity, which could lead to unchecked CD4^+^ and CD8^+^ T cells and thereby results into melanocytes' destruction contributing to GV pathogenesis, progression, and severity. (b) Upon calcium treatment, *ITPR1* mRNA expression is increased which may lead to intracellular Treg calcium influx and calcium-NFAT signalling pathway, thereby results into increased *GZMB* and *SERPINB9* transcripts, leading to increased Treg suppressive capacity. The increased Treg suppressive capacity controls the CD8^+^ and CD4^+^ T cells proliferation and IFN-*γ* production and thereby contributes to melanocytes survival.

Inositol 1,4,5-trisphosphate receptor type 1 (ITPR1) governs the release of calcium from the endoplasmic reticulum [22]. The exact role of ITPR1 in Treg function in GV is unknown. However, upon TCR stimulations in T cells, ITPR1 controls the release of stored calcium from the endoplasmic reticulum [33]. Our previous study has suggested that optimal calcium levels in Tregs are a prerequisite for NFATc1 activation in Treg of GV patients [32]. In current study, we found significantly reduced *ITPR1* transcripts in GV patients. Moreover, our study suggested the role of decreased *ITPR1* transcripts in GV progression and disease severity. Thus, the reduced *ITPR1* expression in Tregs may lead to reduced intracellular Treg calcium levels, resulting in impaired NFAT signalling pathway (Figure 6). Finally, the impaired NFAT signalling pathway may lead to decreased downstream immunosuppressive molecules leading to impaired Treg suppressive function.

As altered *ITPR1* transcripts may lead to impaired Treg activation and suppressive function, we assessed the mRNA expression of Treg-associated suppressive molecules *GZMB*, *NRP1*, *PDCD1*, *FASLG*, and *TNFRS18* in GV patients. Studies have suggested that Tregs control the immune response by granzyme B-dependent cytotoxicity [34]. Upon TCR stimulation and receptor activation, Treg cells produce granzyme B, and previous studies have found increased CD107a expression upon Treg activation, suggesting extracellular degranulation of granzyme B [21, 35]. However, our study revealed significantly reduced *GZMB* transcripts in GV patients. Moreover, we found that the reduced *GZMB* transcripts were associated with GV disease severity and disease progression, suggesting the crucial role of *GZMB* expression in Tregs function. Furthermore, we found a positive correlation between *GZMB* transcripts and *ITPR1* transcripts. Therefore, our study suggests that impaired *ITPR1* transcripts might lead to reduced Treg intracellular calcium levels leading to impaired NFAT signalling pathway in GV (Figure 6). Additionally, our previous study has suggested altered calcium NFAT signalling pathway in GV Tregs [36]. Although the role of NFAT signalling pathway in granzyme B production is unknown, interestingly, studies have suggested for NFAT-binding sites on *GZMB* promoter [36]. Therefore, the decreased *ITPR1* transcripts could further lead to impaired calcium-NFAT signalling pathway, resulting in decreased *GZMB* transcripts, and thus lead to impaired Tregs suppressive function in GV (Figure 6). However, future studies must explore the role of *ITPR1* and NFAT signalling pathway in granzyme B expression. Furthermore, single-nucleotide polymorphisms (SNPs) and epigenetic changes in *GZMB* promoter must be explored as they might be responsible for the decreased *GZMB* expression.

Next, we evaluated the expression levels of Treg suppressive molecule neuropolin 1 (NRP-1). NRP-1 is constitutively expressed on the surface of Tregs and mediates prolonged binding with immature dendritic cells [19, 37]. Anti-NRP-1 antibodies have been shown to abrogate Treg immunosuppressive function [38]. Additionally, lack of NRP-1 on surface of Tregs has been linked with impaired Treg suppressive function and worsening of experimental autoimmune encephalomyelitis severity [38]. However, the role of NRP-1 in GV is unknown. Interestingly, we found significantly reduced *NRP1* transcripts in GV patients, suggesting for the crucial role of *NRP1* in reduced Treg suppressive function leading to GV pathogenesis. Moreover, we found a positive correlation of *NRP1* with *ITPR1* transcripts. Although the link between *ITPR1-*calcium*-*NFAT signalling pathway and *NRP1* is unknown, previous studies suggest that *NRP1* expression is accompanied with high levels of NFATc1 transcript expression [39]. Additionally, NRP-1 expression is controlled by T cells activation, and inhibition of NFATs has shown to suppress NRP-1 expression in Tregs [39]. Overall, our results suggest that the altered *ITPR1-*calcium*-*NFAT signalling pathway might be involved in reduced *NRP1* transcripts which led to impaired Treg suppressive function in GV patients (Figure 6). However, future studies must explore the involvement of *ITPR1-*calcium*-*NFAT signalling pathway, promoter SNPs, and epigenetic changes in *NRP1* promoter, as they might be responsible for decreased *NRP1* expression.

Furthermore, we assessed the expression levels of *SERPINB9* in GV Tregs. *SERPINB9* is an endogenous granzyme B inhibitor [21, 40]. After Treg activation, there is an increase in the production of granzyme B and its endogenous inhibitor *SERPINB9* [21]. Previous studies have shown an increase in granzyme B-mediated apoptosis in SERPINB9 knockout mice, suggesting that Tregs upon activation can undergo self-inflicted apoptosis mediated by granzyme B in absence of SERPINB9 [21]. Interestingly, our study suggested significantly reduced *SERPINB9* transcripts in GV Tregs. Moreover, we found a significant association of *SERPINB9* transcripts with GV disease severity and disease progression. Additionally, previous studies have suggested significantly decreased Treg cells in vitiligo patients [6, 14, 15], suggesting that the increased *SERPINB9* transcripts can lead to Treg cells apoptosis, resulting into decreased Tregs number, thereby contributing to GV pathogenesis, progression, and severity. However, future studies must confirm these findings by studying granzyme caspases-mediated apoptosis pathway in Tregs of GV patients.

Next, we aimed to study the key Treg-associated transcription factors (*GATA2*, *GATA3*, *RUNX1*, *STAT3*, and *STAT5)* in GV patients. The GATA transcription factors are zinc finger motif DNA-binding proteins, and they have a crucial role in Treg function [26, 27]. Genetic ablation in GATA3 has been associated with inflammatory disorder in mice [26]. Additionally, mutations in GATA2 have been associated with autoimmune hepatitis [27]. Additionally, *RUNX1* is a runt-related transcription factor, and it plays a crucial role in generation and function of Treg cells [25]. Moreover, STAT transcription factors, i.e., signal transducers of activation of transcription, control Treg cells development [24, 41]. Genetic knockdown of *STAT3* decreases the Tregs generation [24]. Moreover, *STAT5* plays a crucial role in sustaining FOXP3 expression in Tregs [41]. However, we did not find an association for Treg-associated transcription factors (*GATA2*, *GATA3*, *RUNX1*, *STAT3*, and *STAT5*) with GV pathogenesis, progression, and severity.

Previously, we had studied the *in vitro* Treg suppression assay in GV patients [6]. Our study had revealed significantly decreased *in vitro* Treg-mediated suppression of CD4^+^ and CD8^+^ T cells in GV patients [6]. To study the role of *ITPR1*, *GZMB*, *NRP1*, and *SERPINB9* on *in vitro* Treg suppressive capacity, we carried out correlation analysis. Interestingly, we found a positive correlation for Treg-mediated suppression of CD4^+^ and CD8^+^ T cells with *ITPR1*, *GZMB*, *NRP1*, and *SERPINB9* transcripts in GV patients' Tregs. Therefore, our results suggest that decreased *ITPR1*, *GZMB*, *NRP1*, and *SERPINB9* transcripts might impair Treg suppressive function, resulting in widespread activation of CD4^+^ and CD8^+^ T cells, which could lead to GV pathogenesis (Figure 6).

Our previous study suggested that calcium treatment of Tregs increased the intracellular calcium influx in Tregs of GV patients, due to increased expression of calcium ion channel gene *ORAI1* after calcium treatment [42]. Moreover, the optimum calcium influx enhanced the calcineurin and NFATc1 activity in calcium treated Tregs, which led to increased Treg suppressive capacity [32]. As *ITPR1* is crucial for the release of calcium in Tregs, we studied the transcript levels of *ITPR1* in calcium-treated Tregs of GV patients and controls. Interestingly, our study suggested an increase in mRNA expression of *ITPR1* in Tregs after the calcium treatment (Figure 3(d)). As the increased expression of *ITPR1* after the calcium treatment may further lead to enhanced Treg suppressive activity, we accessed the transcript levels of *GZMB* and *SERPINB9* in calcium-treated Tregs of GV patients. Our study suggested that the calcium treatment increased the expression levels of *GZMB* and *SERPINB9* in GV Tregs (Figures 2(a) and 3(a)). Our results are in line with the previous findings which suggest that calcium signalling modulates cytolytic T lymphocyte function [42]. Overall, our study suggests that calcium treatment may improve Treg suppressive capacity, and targeting the Ca^2+^-calmodulin-calcineurin-NFAT signalling pathway may be a potent therapeutic target for GV.

## 5. Conclusions

Overall, our results for the first time suggest the crucial involvement of *GZMB*, *NRP1*, *SERPINB9*, and *ITPR1* transcripts in reduced Treg-mediated suppression of CD4^+^ and CD8^+^ T cells which lead to GV pathogenesis, progression, and severity. Moreover, our study highlighted that *ITPR1* might be responsible for decreased *GZMB* and *NRP1* transcripts in GV Tregs. Furthermore, our study revealed that the increased *SERPINB9* transcripts might result in endogenous granzyme B-mediated Tregs apoptosis, and the Treg suppressive capacity can be enhanced after calcium treatment in Tregs. These findings may aid in development of Treg-based therapeutics for GV; however, *in vivo* studies must be carried out to validate the role of *GZMB*, *NRP1*, *SERPINB9*, and *ITPR1* in Treg-mediated GV pathogenesis.

## Figures and Tables

**Table 1:** Demographic characteristics of generalized vitiligo (GV) patients and controls.

	GV patients (*n* = 52)	Controls (*n* = 48)
Average age (mean age ± SD)	37.04 ± 11.22 years	32.18 ± 3.18 years
Gender		
Male	28 (53.84%)	26 (54.16%)
Female	24 (46.16%)	22 (45.83%)
Age of onset (mean age ± SD)	18.23 ± 4.21 years	NA
Duration of disease (mean ± SD)	7.22 ± 4.24 years	NA
Extent of disease		
VASI score (mean ± SD)	58.12% ± 22.58%	NA
10–25% VASI (mild GV)	12 (23.07%)	
25–50% VASI (moderate GV)	18 (34.61%)	
50–75% VASI (severe GV)	22 (42.30%)	
Disease activity		
Active vitiligo	28 (54.00%)	NA
Stable vitiligo	24 (46.00%)	
Family history	20 (34.61%)	NA

## Data Availability

Data available on request.

## Supplementary Materials

Supplementary 1 Table S1. Primer sequence for transcript analysis of GZMB, NRP1, ITPR1, SERPINB9, PDCD1, FASLG, UBASH3A, IKZF4, GATA2, GATA3, TNFRSF18, RUNX1, STAT3, and STAT5 genes by qPCR.

Supplementary 2 Figure S1. Melt curve analysis for *GZMB*, *NRP1*, *ITPR1*, *SERPINB9*, *PDCD1*, *FASLG*, *UBASH3A*, and *IKZF4* transcripts. (a) Representative image for melt curve analysis for *GZMB* transcripts. (b) Representative image for melt curve analysis for *NRP1* transcripts. (c) Representative image for melt curve analysis for *ITPR1* transcripts. (d) Representative image for melt curve analysis for *SERPINB9* transcripts. (e) Representative image for melt curve analysis for *PDCD1* transcripts. (f) Representative image for melt curve analysis for *FASLG* transcripts. (g) Representative image for melt curve analysis for *UBASH3A* transcripts and (h) Representative image for melt curve analysis for *IKZF4* transcripts. Figure S2: Melt curve analysis for *GATA2*, *GATA3*, *TNFRSF18*, *RUNX1*, *STAT3*, *STAT5*, and *GAPDH* transcripts. (a) Representative image for melt curve analysis for *GATA2* transcripts. (b) Representative image for melt curve analysis for *GATA3* transcripts. (c) Representative image for melt curve analysis for *TNFRSF18* transcripts. (d) Representative image for melt curve analysis for *RUNX1* transcripts. (e) Representative image for melt curve analysis for *STAT3* transcripts. (f) Representative image for melt curve analysis for *STAT5* transcripts. (g) Representative image for melt curve analysis for *GAPDH* transcripts. Figure S3. Standard curve for estimation of calcium levels: standard curve for calcium ELISA. Figure S4. *PDCD1*, *FASLG*, and *TNFRS18* transcripts in Tregs of GV patients and controls. *PDCD1*, *FASLG*, and *TNFRS18* transcripts in Tregs of 52 GV patients and 48 controls were analyzed by nonparametric Mann–Whitney *U* test. (a) *PDCD1* transcripts in GV, SV, and AV vs controls' Tregs (*p* = 0.4625, *p* = 0.2563, and *p* = 0.3627, respectively). *PDCD1* transcripts in AV vs SV Tregs (*p* = 0.1273). (b) *PDCD1* transcripts in severe GV (50-75% VASI) vs mild GV (10-25% VASI) Tregs (*p* = 0.2427). *PDCD1* transcripts in moderate GV (25-50% VASI) vs mild GV (10-25% VASI) and severe GV (50-75% VASI) Tregs (*p* = 0.1225 and *p* = 0.1423, respectively). (c) There were 0.88-, 0.87-, and 0.98-fold changes in *PDCD1* transcripts for GV, SV, and AV Tregs when compared to controls. (d) *FASLG* transcripts in GV, SV, and AV vs controls' Tregs (*p* = 0.753, *p* = 0.999, and *p* = 0.213, respectively). *FASLG* transcripts in AV vs SV Tregs (*p* = 0.521). (e) *FASLG* transcripts in severe GV (50-75% VASI) vs mild GV (10-25% VASI) Tregs (*p* = 0.113). *FASLG* transcripts in moderate GV (25-50% VASI) vs mild GV (10-25% VASI) and severe GV (50-75% VASI) Tregs (*p* = 0.7123 and *p* = 0.2321, respectively). (f) There were 0.72-, 0.65-, and 0.77-fold changes in *FASLG* transcripts for GV, SV, and AV Tregs as compared to controls. (g) *TNFRS18* transcripts in GV, SV, and AV vs controls' Tregs (*p* = 0.3362, *p* = 0.1272, and *p* = 0.3627, respectively). *TNFRS18* transcripts in AV vs SV Tregs (*p* = 0.1265). (h) *TNFRS18* transcripts in severe GV (50-75% VASI) vs mild GV (10-25% VASI) Tregs (*p* = 0.4653). *TNFRS18* transcripts in moderate GV (25-50% VASI) vs mild GV (10-25% VASI) and severe GV (50-75% VASI) Tregs (*p* = 0.1263 and *p* = 0.7625, respectively). (i) There were 0.86-, 0.87-, and 0.85-fold changes in *TNFRS18* transcripts for GV, SV, and AV Tregs as compared to controls. Figure S5. *UBASH3A*, *GATA2*, and *GATA3* transcripts in Tregs of GV patients and controls. *UBASH3A*, *GATA2*, and *GATA3* transcripts in Tregs of 52 GV patients and 48 controls were analyzed by nonparametric Mann–Whitney *U* test. (a) *UBASH3A* transcripts in GV, SV, and AV vs controls' Tregs (*p* = 0.145, *p* = 0.127, and *p* = 0.647, respectively). *UBASH3A* transcripts in AV vs SV Tregs (*p* = 0.487). (b) *UBASH3A* transcripts in severe GV (50-75% VASI) vs mild GV (10-25% VASI) Tregs (*p* = 0.2347). *UBASH3A* transcripts in moderate GV (25-50% VASI) vs mild GV (10-25% VASI) and severe GV (50-75% VASI) Tregs (*p* = 0.1252 and *p* = 0.1625, respectively). (c) There was a 0.92-, 0.89-, and 1.1-fold changes in *UBASH3A* transcripts for GV, SV, and AV Tregs as compared to controls. (d) *GATA2* transcripts in GV, SV, and AV vs controls' Tregs (*p* = 0.1537, *p* = 0.1922, and *p* = 0.1189, respectively). *GATA2* transcripts in AV vs SV Tregs (*p* = 0.7891). (e) *GATA2* transcripts in severe GV (50-75% VASI) vs mild GV (10-25% VASI) Tregs (*p* = 0.1922). *GATA2* transcripts in moderate GV (25-50% VASI) vs mild GV (10-25% VASI) and severe GV (50-75% VASI) Tregs (*p* = 0.7654 and *p* = 0.1165, respectively). (f) There was a 0.89-, 0.86-, and 0.84-fold changes in *GATA2* transcripts for GV, SV, and AV Tregs as compared to controls. (g) *GATA3* transcripts in GV, SV, and AV vs controls' Tregs (*p* = 0.2224, *p* = 0.86322, and *p* = 0.1152, respectively). *GATA3* transcripts in AV vs SV Tregs (*p* = 0.3641). (h) *GATA3* transcripts in severe GV (50-75% VASI) vs mild GV (10-25% VASI) Tregs (*p* = 0.8742). *GATA3* transcripts in moderate GV (25-50% VASI) vs mild GV (10-25% VASI) and severe GV (50-75% VASI) Tregs (*p* = 0.1645 and *p* = 0.8873, respectively). (i) There was a 0.89-, 0.92-, and 0.97-fold changes in *GATA3* transcripts for GV, SV, and AV Tregs as compared to controls. Figure S6. *RUNX1*, *STAT3*, and *STAT5* transcripts in Tregs of GV patients and controls. *RUNX1*, *STAT3*, and *STAT5* transcripts in Tregs of 52 GV patients and 48 controls were analyzed by nonparametric Mann–Whitney *U* test. (a) *RUNX1* transcripts in GV, SV, and AV vs controls' Tregs (*p* = 0.1152, *p* = 0.1254, and *p* = 0.1455, respectively). *RUNX1* transcripts in AV vs SV Tregs (*p* = 0.2235). (b) *RUNX1* transcripts in severe GV (50-75% VASI) vs mild GV (10-25% VASI) Tregs (*p* = 0.1455). *RUNX1* transcripts in moderate GV (25-50% VASI) vs mild GV (10-25% VASI) and severe GV (50-75% VASI) Tregs (*p* = 0.1152 and *p* = 0.1164, respectively). (c) There was a 0.1.1-, 1.2-, and 0.97-fold changes in *RUNX1* transcripts for GV, SV, and AV Tregs when compared to controls. (d) *STAT3* transcripts in GV, SV, and AV vs controls' Tregs (*p* = 0.2333, *p* = 0.2143, and *p* = 0.3541, respectively). *STAT3* transcripts in AV vs SV Tregs (*p* = 0.3436). (e) *STAT3* transcripts in 50-75% severe GV vs 10-25% mild GV Tregs (*p* = 0.4222). *STAT3* transcripts in moderate GV (25-50% VASI) vs mild GV (10-25% VASI) and severe GV (50-75% VASI) Tregs (*p* = 0.1455 and *p* = 0.8423, respectively). (f) There was a 1.01-, 1.3-, and 1.0-fold changes in *STAT3* transcripts for GV, SV, and AV Tregs as compared to controls. (g) *STAT5* transcripts in GV, SV, and AV vs controls' Tregs (*p* = 0.1454, *p* = 0.2241, and *p* = 0.1535, respectively). *STAT5* transcripts in AV vs SV Tregs (*p* = 0.4535). (h) *STAT5* transcripts in severe GV (50-75% VASI) vs mild GV (10-25% VASI) Tregs (*p* = 0.2532). *STAT5* transcripts in moderate GV (25-50% VASI) vs mild GV (10-25% VASI) and severe GV (50-75% VASI) Tregs (*p* = 0.1432 and *p* = 0.1484, respectively). (i) There was a 0.99-, 1.1-, and 0.94-fold changes in *STAT5* transcripts for GV, SV, and AV Tregs as compared to controls. Figure S7. Age of onset analysis for *GZMB*, *NRP1*, P*DCD1*, *FASLG*, *TNFRS18*, *SERPINB9*, and *ITPR1* transcripts in GV Tregs and control Tregs. (a) *GZMB* transcript levels in Tregs of different age of onset groups (1-20 vs 21-40 years: *p* = 0.1263; 1-20 vs 41-60 years: *p* = 0.6532; 21-40 vs 41-60 years: *p* = 0.9836). (b) *NRP1* transcript levels in Tregs of different age of onset groups (1-20 vs 21-40 years: *p* = 0.1563; 1-20 vs 41-60 years: *p* = 0.8832; 21-40 vs 41-60 years: *p* = 0.6863). (c) *PDCD1* transcript levels in Tregs of different age of onset groups (1-20 vs 21-40 years: *p* = 0.1348; 1-20 vs 41-60 years: *p* = 0.2842; 21-40 vs 41-60 years:*p* = 0.4923). (d) *FASLG* transcript levels in Tregs of different age of onset groups (1-20 vs 21-40 years: *p* = 0.1323; 1-20 vs 41-60 years: *p* = 0.2932; 21-40 vs 41-60 years: *p* = 0.1937). (e) *TNFRS18* transcript levels in Tregs of different age of onset groups (1-20 vs 21-40 years:*p* = 0.4627; 1-20 vs 41-60 years: *p* = 0.2847, 21-40 vs 41-60 years: *p* = 0.2948). (f) *SERPINB9* transcript levels in Tregs of different age of onset groups (1-20 vs 21-40 years: *p* = 0.3533; 1-20 vs 41-60 years: *p* = 0.4242; 21-40 vs 41-60 years: *p* = 0.4363). (g) *ITPR1* transcript levels in Tregs of different age of onset groups (1-20 vs 21-40 years: *p* = 0.2648; 1-20 vs 41-60 years: *p* = 0.3762; 21-40 vs 41-60 years: *p* = 0.2635). Figure S8. Age of onset analysis for *UBASH3A*, *GATA2*, *GATA3*, *RUNX1*, *STAT3*, and *STAT5* transcripts in GV Tregs and control Tregs. (a) *UBASH3A* transcript levels in Tregs of different age of onset groups (1-20 vs 21-40 years: *p* = 0.4836; 1-20 vs 41-60 years: *p* = 0.5247; 21-40 vs 41-60 years: *p* = 0.1938). (b) *GATA2* transcript levels in Tregs of different age of onset groups (1-20 vs 21-40 years: *p* = 0.1321; 1-20 vs 41-60 years: *p* = 0.1380; 21-40 vs 41-60 years: *p* = 0.5221). (c) *GATA3* transcript levels in Tregs of different age of onset groups (1-20 vs 21-40 years: *p* = 0.1241; 1-20 vs 41-60 years: *p* = 0.5384; 21-40 vs 41-60: *p* = 0.2411). (d) *RUNX1* transcript levels in Tregs of different age of onset groups (1-20 vs 21-40 years: *p* = 0.1434; 1-20 vs 41-60 years: *p* = 0.3210; 21-40 vs 41-60 years: *p* = 0.1281). (e) *STAT3* transcript levels in Tregs of different age of onset groups (1-20 vs 21-40 years: *p* = 0.4826; 1-20 vs 41-60 years: *p* = 0.3292; 21-40 vs 41-60 years: *p* = 0.1196). (f) *STAT5* transcript levels in Tregs of different age of onset groups (1-20 vs 21-40 years: *p* = 0.1284; 1-20 vs 41-60 years: *p* = 0.3421; 21-40 vs 41-60 years: *p* = 0.1248). Figure S9. Gender-based analysis for *GZMB*, *NRP1*, *PDCD1*, *FASLG*, *TNFRS18*, *SERPINB9*, and *ITPR1* transcripts in GV Tregs and control Tregs. (a) *GZMB* transcript levels in Tregs of male and female GV patients (*p* = 0.2462). (b) *NRP1* transcript levels in Tregs of male and female GV patients (*p* = 0.1739). (c) *PDCD1* transcript levels in Tregs of male and female GV patients (*p* = 0.5642). (d) *FASLG* transcript levels in Tregs of male and female GV patients (*p* = 0.4852). (e) *TNFRS18* transcript levels in Tregs of male and female GV patients (*p* = 0.6737). (f) *SERPINB9* transcript levels in Tregs of male and female GV patients (*p* = 0.5742). (g) *ITPR1* transcript levels in Tregs of male and female GV patients (*p* = 0.4513). Figure S10. Gender-based analysis for *UBASH3A*, *GATA2*, *GATA3*, *RUNX1*, *STAT3*, and *STAT5* transcripts in GV Tregs and control Tregs. (a) *UBASH3A* transcript levels in Tregs of male and female GV patients (*p* = 0.4547). (b) *GATA2* transcript levels in Tregs of male and female GV patients (*p* = 0.6780). (c) *GATA3* transcript levels in Tregs of male and female GV patients (*p* = 0.6734). (d) *RUNX1* transcript levels in Tregs of male and female GV patients (*p* = 0.6797). (e) *STAT3* transcript levels in Tregs of male and female GV patients (*p* = 0.6792). (f) *STAT5* transcript levels in Tregs of male and female GV patients (*p* = 0.7621).
