# Supplementary material for: Decreased GZMB, NRP1, ITPR1, and SERPINB9 Transcripts Lead to Reduced Regulatory T Cells Suppressive Capacity in Generalized Vitiligo Patients
Source: J Immunol Res. 2022 Sep 15;2022:3426717. doi: 10.1155/2022/3426717 (PMC9500245; doi:10.1155/2022/3426717)
Supplement: Supplementary 1 — Table S1. Primer sequence for transcript analysis of GZMB, NRP1, ITPR1, SERPINB9, PDCD1, FASLG, UBASH3A, IKZF4, GATA2, GATA3, TNFRSF18, RUNX1, STAT3, and STAT5 genes by qPCR. [file 3426717.f1.doc]

| **Table S1. Primer sequence for transcript analysis of *GZMB, NRP1, ITPR1, SERPINB9, PDCD1, FASLG, UBASH3A, IKZF4, GATA2, GATA3, TNFRSF18, RUNX1, STAT3* and *STAT5* genes by qPCR.** | | | |
| --- | --- | --- | --- |
| **Gene** | **Primer** | **Primer sequence** | **Annealing temperature (^0^C)** |
| ***GZMB***  **(Granzyme B)** | Forward Primer | 5’ TTAAGGGGGACTCTGGAGG 3’ | 60 |
|  | Reverse Primer | 5’ CGTCCATAGGAGACAATGC 3’ |  |
| ***SERPINB9***  **(Serpin family B member 9)** | Forward Primer | 5’ AGATGGCCCAGGCACTGTC 3’ | 57 |
|  | Reverse Primer | 5’ TCCTCTGTCCAACGGGGC 3’ |  |
| ***UBASH3A***  **(Ubiquitin Associated and SH3 Domain Containing A)** | Forward Primer | 5’ TGGCCTGGCTGCATGATC 3’ | 55 |
|  | Reverse Primer | 5’ GCCCCGTTGGACAGAGGA 3’ |  |
| ***FASLG***  **(Fas ligand)** | Forward Primer | 5’ GCCCATTTAACAGGCAAGTCC 3’ | 60 |
|  | Reverse Primer | 5’ GCAGGACAATTCCATAGGTGTC 3’ |  |
| ***ITPR1***  **(Inositol 1,4,5-trisphosphate receptor type 1)** | Forward Primer | 5’ GGGCCTGGTTGATGATCG 3’ | 58 |
|  | Reverse Primer | 5’ GGCAGAGTAGCGGTTCATG 3’ |  |
| ***NRP1***  **(Neuropilin-1)** | Forward Primer | 5’ TGTTGGCCCTCACATTGGG 3’ | 60 |
|  | Reverse Primer | 5’ GAGACACTGCTCTGCAAGAC 3’ |  |
| ***PDCD1***  **(Programmed cell death protein 1)** | Forward Primer | 5’ TGAAGGAGGACCCCTCAGC 3’ | 60 |
|  | Reverse Primer | 5’ CGCCACAGGAAATCCAGC 3’ |  |
| ***TNFRS18***  ***(G*lucocorticoid-induced TNFR-related protein)** | Forward Primer | 5’ GATTACCCGGGCGAGGAG 3’ | 60 |
|  | Reverse Primer | 5’ GACTGTACCCCCTGGCCT 3’ |  |
| ***RUNX1***  **(Runt-related transcription factor 1)** | Forward Primer | 5’ AGTGGAAGAGGGAAAAGC 3’ | 60 |
|  | Reverse Primer | 5’ ATCCACTGTGATTTTGATGG 3’ |  |
| ***STAT3***  **(Signal transducer and activator of transcription 3)** | Forward Primer | 5’ AGAAGGACATCAGCGGTAAG 3’ | 59 |
|  | Reverse Primer | 5’ AGTGGAGACACCAGGATATTG 3’ |  |
| ***STAT5***  **(Signal transducer and activator of transcription 5)** | Forward Primer | 5’ CTCCAGAAAACATATGACCGC 3’ | 60 |
|  | Reverse Primer | 5’ TCATTGTACAGAATGTGCCGG 3’ |  |
| ***GATA2***  **(GATA-binding factor 2)** | Forward Primer | 5’ GTTCAGAAGGCCGGGAGTGTG 3’ | 58 |
|  | Reverse Primer | 5’ ATGAGTGGTCGGTTCTGCCCAT 3’ |  |
| ***GATA3***  **(GATA-binding factor 2)** | Forward Primer | 5’ CACGACACTGTGGCGGAGAAAT 3’ | 60 |
|  | Reverse Primer | 5’ GGCCGGTTCACCTGGTGTAGC 3’ |  |
| ***GAPDH***  **(Glyceraldehyde 3-phosphate dehydrogenase)** | Forward Primer | 5’ CATCACCATCTTCCAGGAGCGAG 3’ | 60 |
|  | Reverse Primer | 5’ CCTGCAAATGAGCCCCAGCCT 3’ |  |
