# Supplementary material for: Decreased GZMB, NRP1, ITPR1, and SERPINB9 Transcripts Lead to Reduced Regulatory T Cells Suppressive Capacity in Generalized Vitiligo Patients
Source: J Immunol Res. 2022 Sep 15;2022:3426717. doi: 10.1155/2022/3426717 (PMC9500245; doi:10.1155/2022/3426717)
Supplement: Supplementary 2 — Figure S1. Melt curve analysis for GZMB, NRP1, ITPR1, SERPINB9, PDCD1, FASLG, UBASH3A, and IKZF4 transcripts. (a) Representative image for melt curve analysis for GZMB transcripts. (b) Representative image for melt curve analysis for NRP1 transcripts. (c) Representative image for melt curve analysis for ITPR1 transcripts. (d) Representative image for melt curve analysis for SERPINB9 transcripts. (e) Representative image for melt curve analysis for PDCD1 transcripts. (f) Representative image for melt curve analysis for FASLG transcripts. (g) Representative image for melt curve analysis for UBASH3A transcripts and (h) Representative image for melt curve analysis for IKZF4 transcripts. Figure S2: Melt curve analysis for GATA2, GATA3, TNFRSF18, RUNX1, STAT3, STAT5, and GAPDH transcripts. (a) Representative image for melt curve analysis for GATA2 transcripts. (b) Representative image for melt curve analysis for GATA3 transcripts. (c) Representative image for melt curve analysis for TNFRSF18 transcripts. (d) Representative image for melt curve analysis for RUNX1 transcripts. (e) Representative image for melt curve analysis for STAT3 transcripts. (f) Representative image for melt curve analysis for STAT5 transcripts. (g) Representative image for melt curve analysis for GAPDH transcripts. Figure S3. Standard curve for estimation of calcium levels: standard curve for calcium ELISA. Figure S4. PDCD1, FASLG, and TNFRS18 transcripts in Tregs of GV patients and controls. PDCD1, FASLG, and TNFRS18 transcripts in Tregs of 52 GV patients and 48 controls were analyzed by nonparametric Mann–Whitney U test. (a) PDCD1 transcripts in GV, SV, and AV vs controls' Tregs (p = 0.4625, p = 0.2563, and p = 0.3627, respectively). PDCD1 transcripts in AV vs SV Tregs (p = 0.1273). (b) PDCD1 transcripts in severe GV (50-75% VASI) vs mild GV (10-25% VASI) Tregs (p = 0.2427). PDCD1 transcripts in moderate GV (25-50% VASI) vs mild GV (10-25% VASI) and severe GV (50-75% VASI) Tregs (p = 0.1225 and p = [file 3426717.f2.doc]

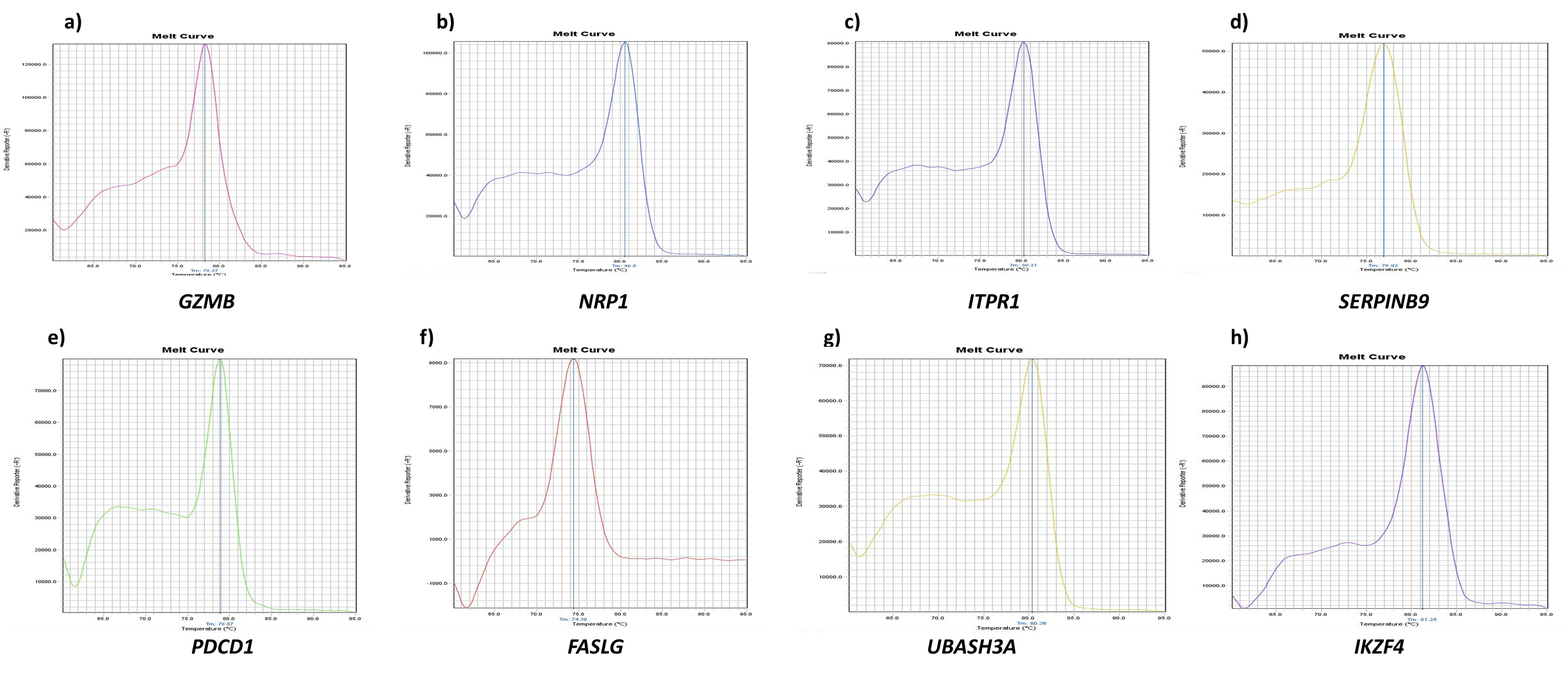


**Figure S1. Melt curve analysis for** ***GZMB, NRP1, ITPR1, SERPINB9,*** ***PDCD1, FASLG, UBASH3A,* and** ***IKZF4* transcripts*.***

a) Representative image for melt curve analysis for *GZMB* transcripts*.* b) Representative image for melt curve analysis for *NRP1* transcripts*.* c) Representative image for melt curve analysis for *ITPR1* transcripts. d) Representative image for melt curve analysis for *SERPINB9* transcripts. e) Representative image for melt curve analysis for *PDCD1* transcripts*.* f) Representative image for melt curve analysis for *FASLG* transcripts. g) Representative image for melt curve analysis for *UBASH3A* transcripts and h) Representative image for melt curve analysis for *IKZF4* transcripts.

**
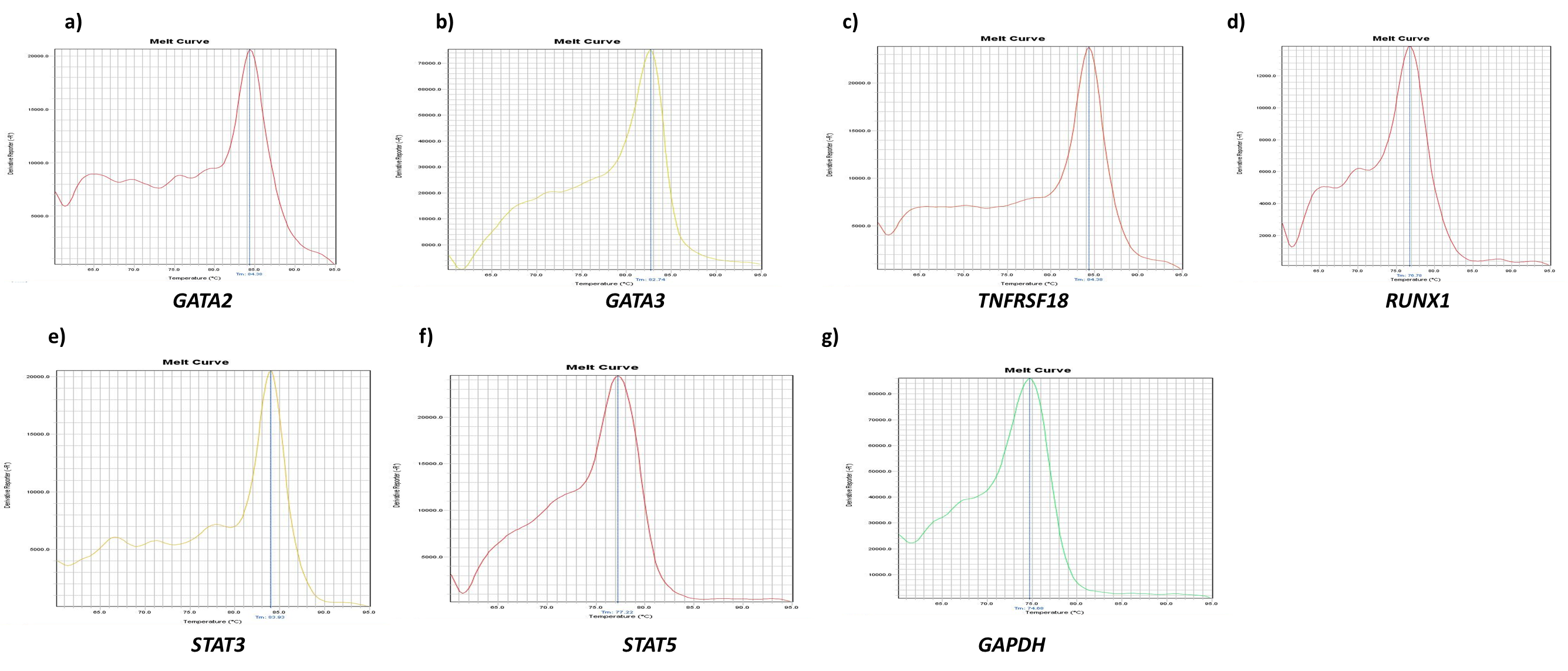
**

**Figure S2: Melt curve analysis for** ***GATA2, GATA3, TNFRSF18, RUNX1, STAT3, STAT5* and *GAPDH* transcripts.** a) Representative image for melt curve analysis for *GATA2* transcripts*.* b) Representative image for melt curve analysis for *GATA3* transcripts*.* c) Representative image for melt curve analysis for *TNFRSF18* transcripts. d) Representative image for melt curve analysis for *RUNX1* transcripts. e) Representative image for melt curve analysis for *STAT3* transcripts*.* f) Representative image for melt curve analysis for *STAT5* transcripts. g) Representative image for melt curve analysis for *GAPDH* transcripts*.*

**

**

**Figure S3. Standard curve for estimation of calcium levels:** Standard curve for calcium ELISA.

**
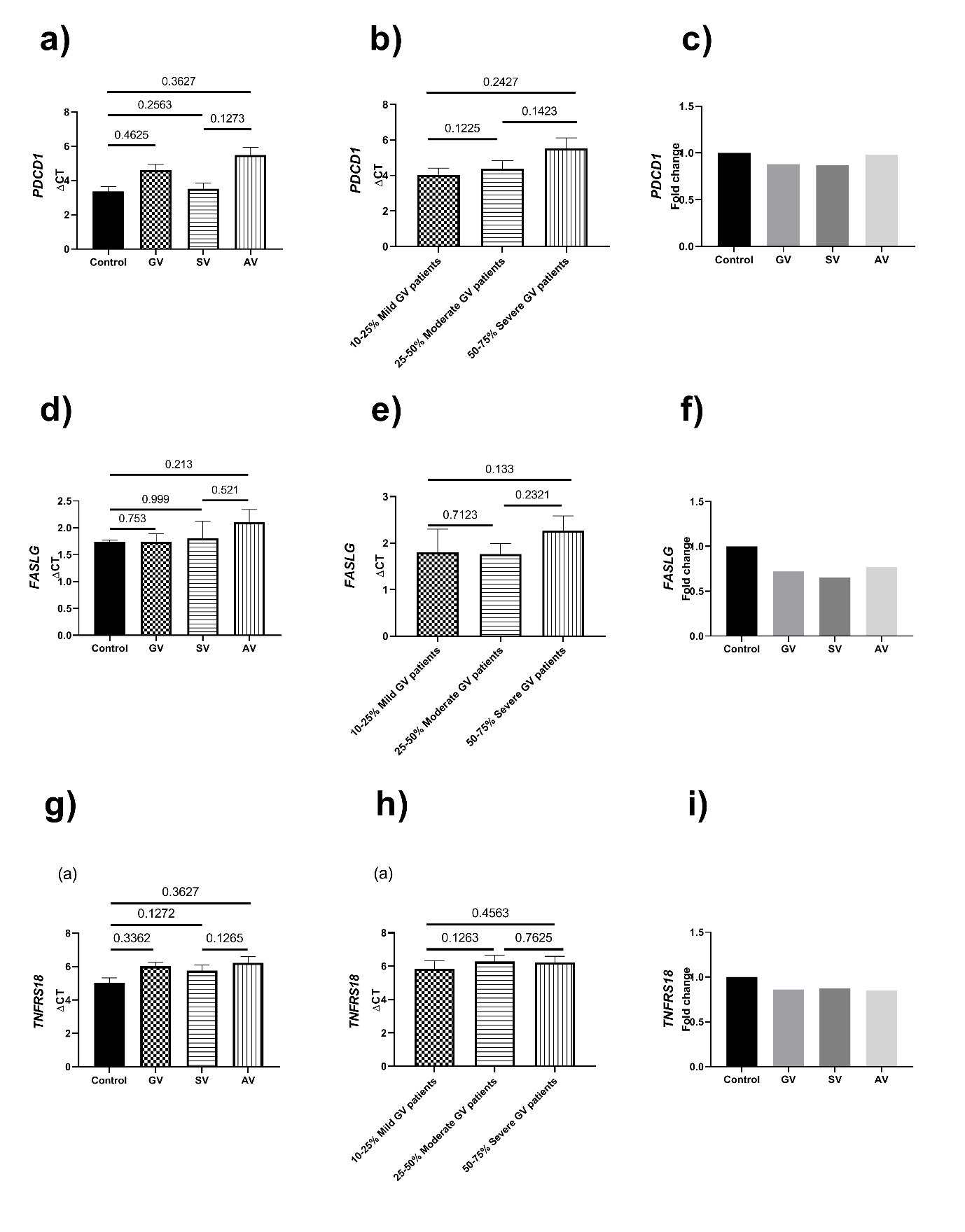
**

**Figure S4. *PDCD1, FASLG* and *TNFRS18* transcripts in Tregs of GV patients and controls.**

*PDCD1, FASLG* and *TNFRS18* transcripts in Tregs of 52 GV patients and 48 controls were analysed by Non-parametric Mann-Whitney U test. a) *PDCD1* transcripts in GV, SV and AV vs controls’ Tregs (p=0.4625, p=0.2563 and p=0.3627, respectively). *PDCD1* transcripts in AV vs SV Tregs (p=0.1273). b) *PDCD1* transcripts in severe GV (50-75% VASI) vs Mild GV (10-25% VASI) Tregs (p=0.2427). *PDCD1* transcripts in Moderate GV (25-50% VASI) vs Mild GV (10-25% VASI) and severe GV (50-75% VASI) Tregs (p=0.1225 & p=0.1423, respectively). c) There was a 0.88, 0.87 and 0.98-fold changes in *PDCD1* transcripts for GV, SV and AV Tregs when compared to controls. d) *FASLG* transcripts in GV, SV and AV vs controls’ Tregs (p=0.753, p=0.999 and p=0.213, respectively). *FASLG* transcripts in AV vs SV Tregs (p=0.521). e) *FASLG* transcripts in severe GV (50-75% VASI) vs Mild GV (10-25% VASI) Tregs (p=0.113). *FASLG* transcripts in Moderate GV (25-50% VASI) vs Mild GV (10-25% VASI) and severe GV (50-75% VASI) Tregs (p=0.7123 & p=0.2321, respectively). f) There was a 0.72, 0.65 and 0.77-fold changes in *FASLG* transcripts for GV, SV and AV Tregs as compared to controls. g) *TNFRS18* transcripts in GV, SV and AV vs controls’ Tregs (p=0.3362, p=0.1272 & p=0.3627, respectively). *TNFRS18* transcripts in AV vs SV Tregs (p=0.1265). h) *TNFRS18* transcripts in severe GV (50-75% VASI) vs Mild GV (10-25% VASI) Tregs (p=0.4653). *TNFRS18* transcripts in Moderate GV (25-50% VASI) vs Mild GV (10-25% VASI) and severe GV (50-75% VASI) Tregs (p=0.1263 & p=0.7625, respectively). i) There was a 0.86, 0.87 and 0.85-fold changes in *TNFRS18* transcripts for GV, SV and AV Tregs as compared to controls.

**
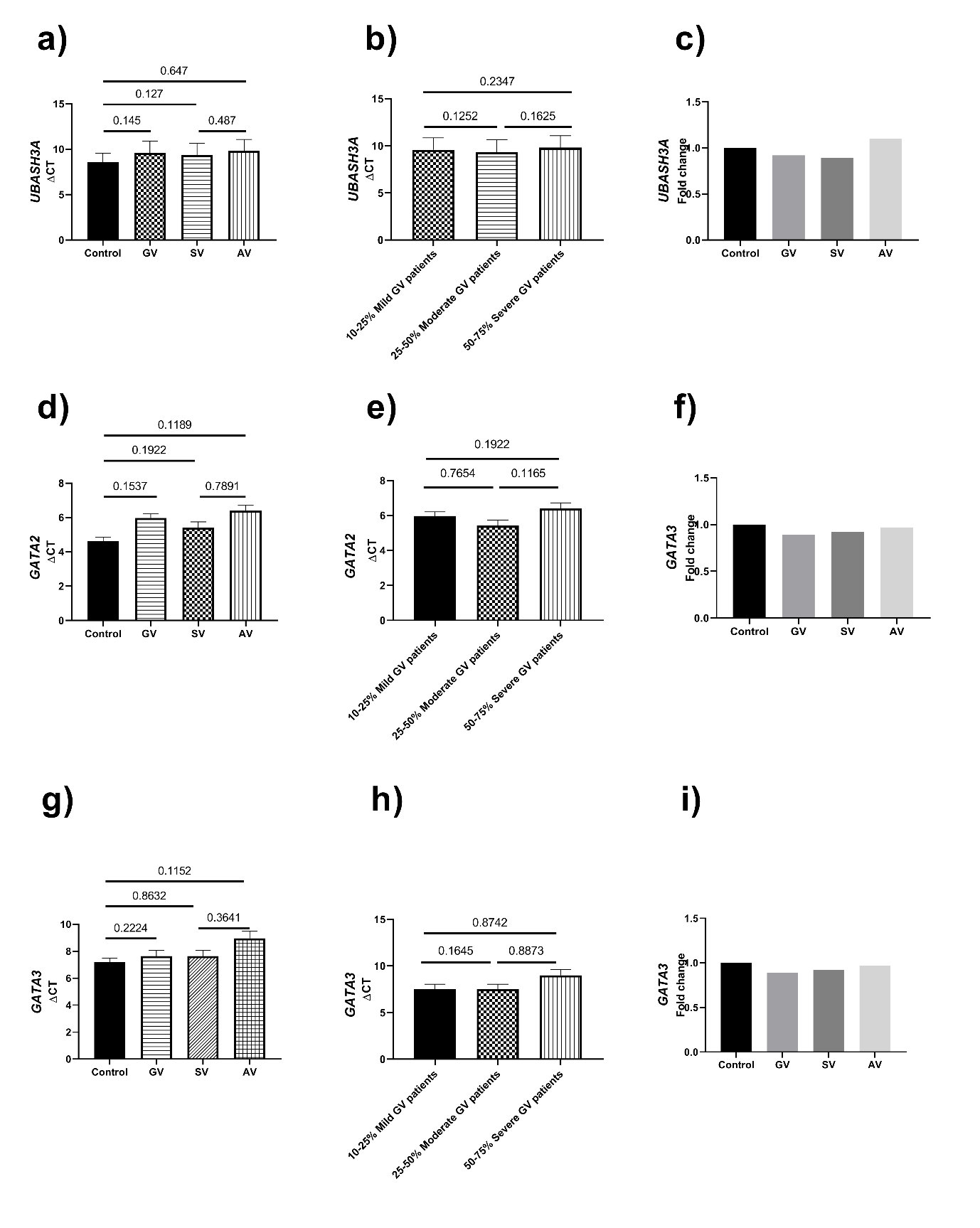
**

**Figure S5. *UBASH3A, GATA2* and *GATA3* transcripts in Tregs of GV patients and controls.**

*UBASH3A, GATA2* and *GATA3* transcripts in Tregs of 52 GV patients and 48 controls were analysed by Non-parametric Mann-Whitney U test. a) *UBASH3A* transcripts in GV, SV and AV vs controls’ Tregs (p=0.145, p=0.127 and p=0.647, respectively). *UBASH3A* transcripts in AV vs SV Tregs (p=0.487). b) *UBASH3A* transcripts in severe GV (50-75% VASI) vs Mild GV (10-25% VASI) Tregs (p=0.2347). *UBASH3A* transcripts in Moderate GV (25-50% VASI) vs Mild GV (10-25% VASI) and severe GV (50-75% VASI) Tregs (p=0.1252 & p=0.1625, respectively). c) There was a 0.92, 0.89 and 1.1-fold changes in *UBASH3A* transcripts for GV, SV and AV Tregs as compared to controls. d) *GATA2* transcripts in GV, SV and AV vs controls’ Tregs (p=0.1537, p=0.1922 & p=0.1189, respectively). *GATA2* transcripts in AV vs SV Tregs (p=0.7891). e) *GATA2* transcripts in severe GV (50-75% VASI) vs Mild GV (10-25% VASI) Tregs (p=0.1922). *GATA2* transcripts in Moderate GV (25-50% VASI) vs Mild GV (10-25% VASI) and severe GV (50-75% VASI) Tregs (p=0.7654 & p=0.1165, respectively). f) There was a 0.89, 0.86 and 0.84-fold changes in *GATA2* transcripts for GV, SV and AV Tregs as compared to controls. g) *GATA3* transcripts in GV, SV and AV vs controls’ Tregs (p=0.2224, p=0.86322 and p=0.1152, respectively). *GATA3* transcripts in AV vs SV Tregs (p=0.3641). h) *GATA3* transcripts in severe GV (50-75% VASI) vs Mild GV (10-25% VASI) Tregs (p=0.8742). *GATA3* transcripts in Moderate GV (25-50% VASI) vs Mild GV (10-25% VASI) and severe GV (50-75% VASI) Tregs (p=0.1645 & p=0.8873, respectively). i) There was a 0.89, 0.92 and 0.97-fold changes in *GATA3* transcripts for GV, SV and AV Tregs as compared to controls.

**
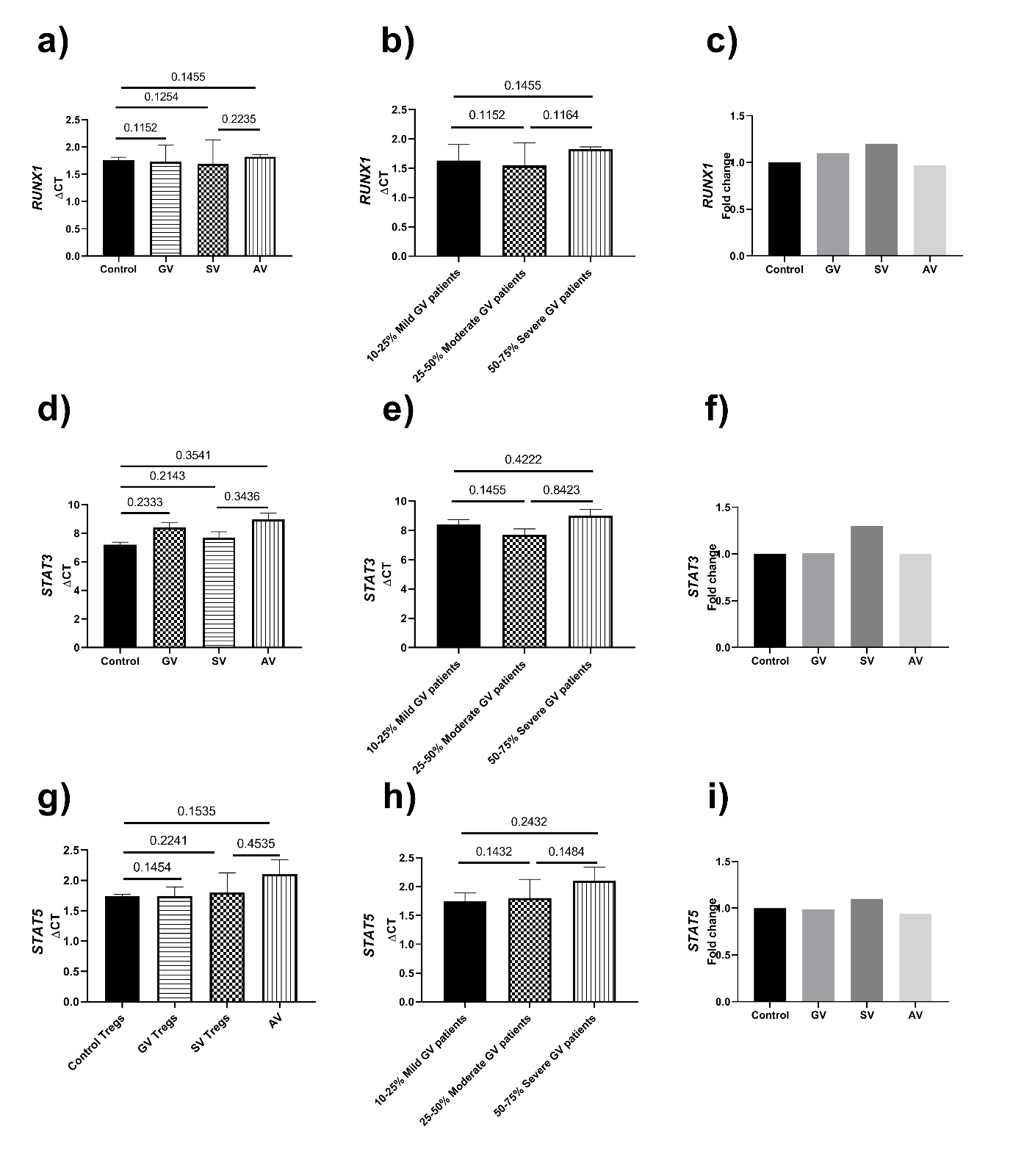
**

**Figure S6. *RUNX1, STAT3* and *STAT5* transcripts in Tregs of GV patients and controls.**

*RUNX1, STAT3* and *STAT5* transcripts in Tregs of 52 GV patients and 48 controls were analysed by Non-parametric Mann-Whitney U test. a) *RUNX1* transcripts in GV, SV and AV vs controls’ Tregs (p=0.1152, p=0.1254 and p=0.1455, respectively). *RUNX1* transcripts in AV vs SV Tregs (p=0.2235). b) *RUNX1* transcripts in severe GV (50-75% VASI) vs Mild GV (10-25% VASI) Tregs (p=0.1455). *RUNX1* transcripts in Moderate GV (25-50% VASI) vs Mild GV (10-25% VASI) and severe GV (50-75% VASI) Tregs (p=0.1152 & p=0.1164, respectively). c) There was a 0.1.1, 1.2 and 0.97-fold changes in *RUNX1* transcripts for GV, SV and AV Tregs when compared to controls. d) *STAT3* transcripts in GV, SV and AV vs controls’ Tregs(p=0.2333, p=0.2143 and p=0.3541, respectively). *STAT3* transcripts in AV vs SV Tregs (p=0.3436). e) *STAT3* transcripts in 50-75% severe GV vs 10-25% Mild GV Tregs (p=0.4222). *STAT3* transcripts in Moderate GV (25-50% VASI) vs Mild GV (10-25% VASI) and severe GV (50-75% VASI) Tregs (p=0.1455 & p=0.8423, respectively). f) There was a 1.01, 1.3 and 1.0-fold changes in *STAT3* transcripts for GV, SV and AV Tregs as compared to controls. g) *STAT5* transcripts in GV, SV and AV vs controls’ Tregs (p=0.1454, p=0.2241 and p=0.1535, respectively). *STAT5* transcripts in AV vs SV Tregs (p=0.4535). h) *STAT5* transcripts in severe GV (50-75% VASI) vs Mild GV (10-25% VASI) Tregs (p=0.2532). *STAT5* transcripts in Moderate GV (25-50% VASI) vs Mild GV (10-25% VASI) and severe GV (50-75% VASI) Tregs (p=0.1432 & p=0.1484, respectively). i) There was a 0.99, 1.1 and 0.94-fold changes in *STAT5* transcripts for GV, SV and AV Tregs as compared to controls.

**
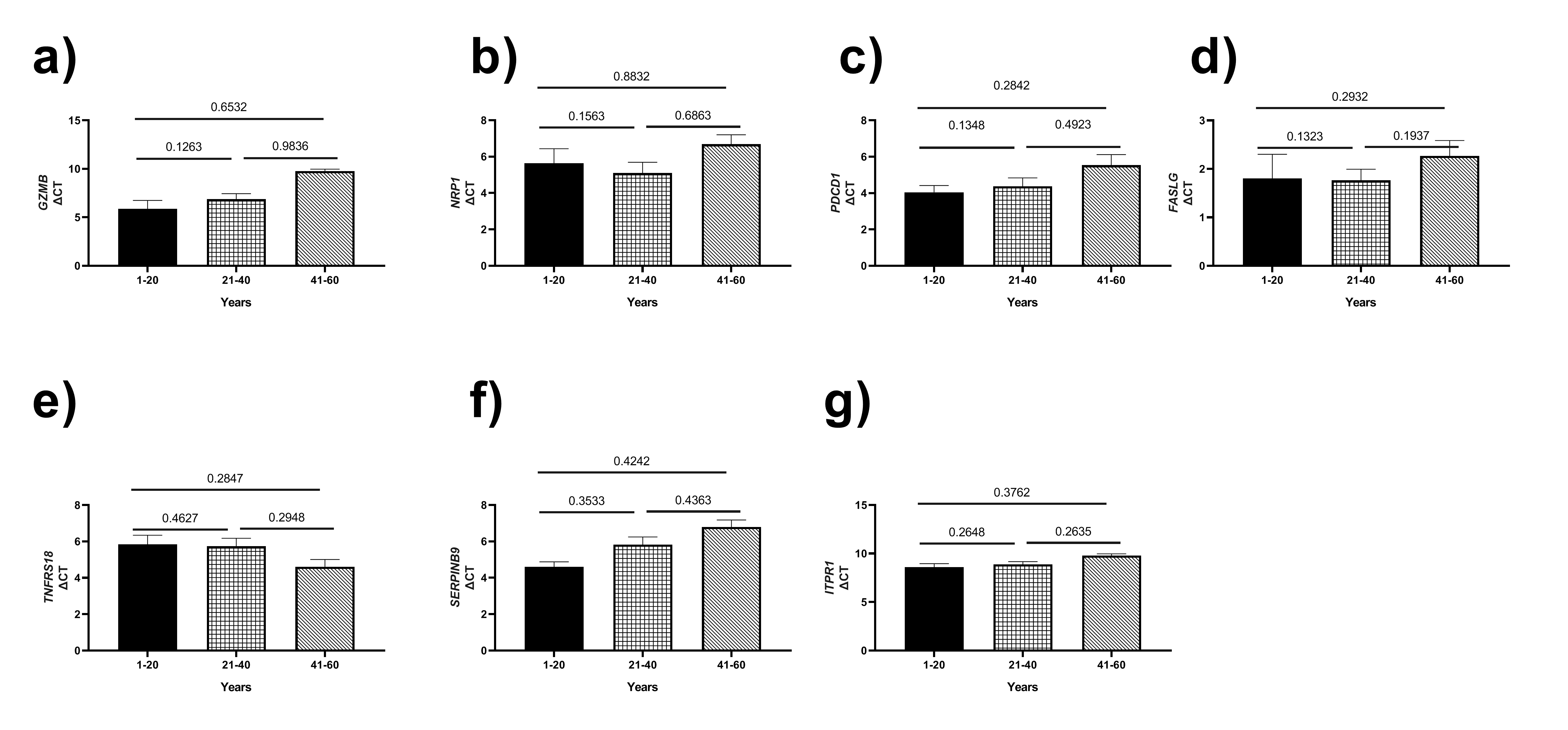
**

**Figure S7. Age of onset analysis for *GZMB, NRP1, PDCD1, FASLG, TNFRS18, SERPINB9* and *ITPR1* transcripts in GV Tregs and control Tregs.**

a) *GZMB* transcript levels in Tregs of different age of onset groups (1-20 vs 21-40 years: p=0.1263; 1-20 vs 41-60 years: p=0.6532; 21-40 vs 41-60 years: p=0.9836). b) *NRP1* transcript levels in Tregs of different age of onset groups (1-20 vs 21-40 years: p=0.1563; 1-20 vs 41-60 years: p=0.8832; 21-40 vs 41-60 years: p=0.6863). c) *PDCD1* transcript levels in Tregs of different age of onset groups (1-20 vs 21-40 years: p=0.1348; 1-20 vs 41-60 years: p=0.2842; 21-40 vs 41-60 years: p=0.4923). d) *FASLG* transcript levels in Tregs of different age of onset groups (1-20 vs 21-40 years: p=0.1323; 1-20 vs 41-60 years: p=0.2932; 21-40 vs 41-60 years: p=0.1937). e) *TNFRS18* transcript levels in Tregs of different age of onset groups (1-20 vs 21-40 years: p=0.4627; 1-20 vs 41-60 years: p=0.2847, 21-40 vs 41-60 years: p=0.2948). f) *SERPINB9* transcript levels in Tregs of different age of onset groups (1-20 vs 21-40 years: p=0.3533; 1-20 vs 41-60 years: p=0.4242; 21-40 vs 41-60 years: p=0.4363)**.** g) *ITPR1* transcript levels in Tregs of different age of onset groups (1-20 vs 21-40 years: p=0.2648; 1-20 vs 41-60 years: p=0.3762; 21-40 vs 41-60 years: p=0.2635).

**
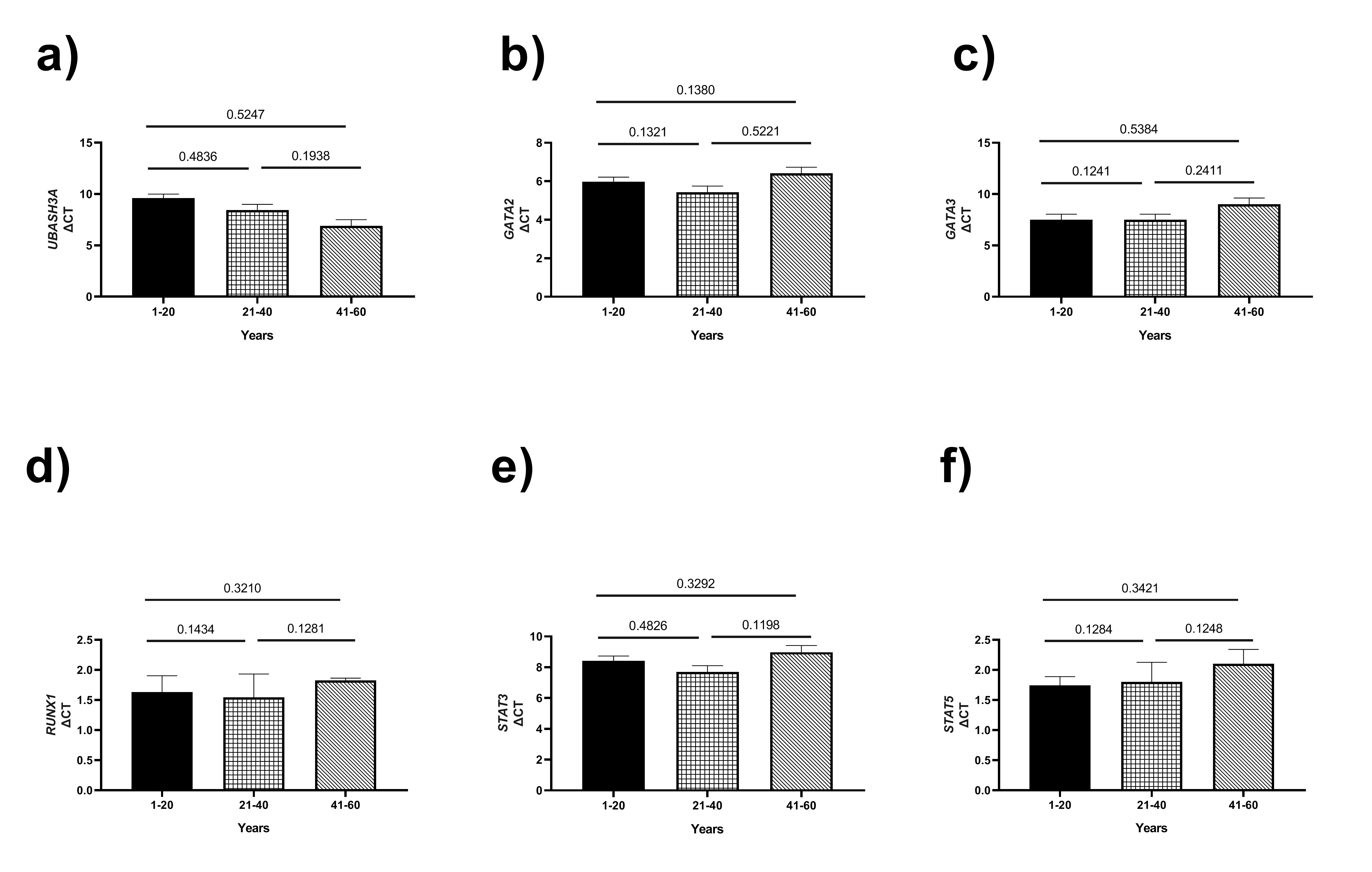
**

**Figure S8. Age of onset analysis for *UBASH3A, GATA2, GATA3, RUNX1, STAT3,* and *STAT5* transcripts in GV Tregs and control Tregs.**

a) *UBASH3A* transcript levels in Tregs of different age of onset groups (1-20 vs 21-40 years: p=0.4836; 1-20 vs 41-60 years: p=0.5247; 21-40 vs 41-60 years: p=0.1938). b) *GATA2* transcript levels in Tregs of different age of onset groups (1-20 vs 21-40 years: p=0.1321; 1-20 vs 41-60 years: p=0.1380; 21-40 vs 41-60 years: p=0.5221). c) *GATA3* transcript levels in Tregs of different age of onset groups (1-20 vs 21-40 years: p=0.1241; 1-20 vs 41-60 years: p=0.5384; 21-40 vs 41-60: p=0.2411). d) *RUNX1* transcript levels in Tregs of different age of onset groups (1-20 vs 21-40 years: p=0.1434; 1-20 vs 41-60 years: p=0.3210; 21-40 vs 41-60 years: p=0.1281). e) *STAT3* transcript levels in Tregs of different age of onset groups (1-20 vs 21-40 years: p=0.4826; 1-20 vs 41-60 years: p=0.3292; 21-40 vs 41-60 years: p=0.1196). f) *STAT5* transcript levels in Tregs of different age of onset groups (1-20 vs 21-40 years: p=0.1284; 1-20 vs 41-60 years: p=0.3421; 21-40 vs 41-60 years: p=0.1248).

**
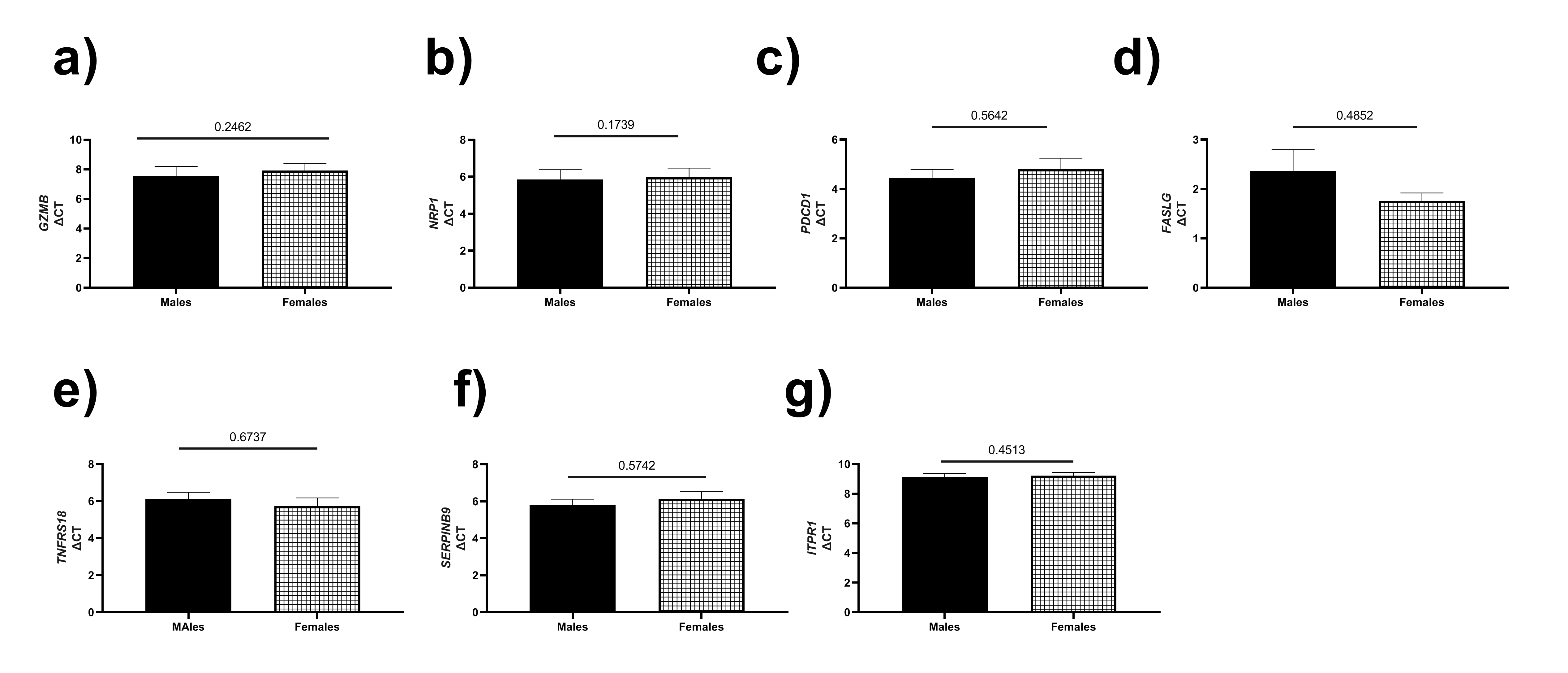
**

**Figure S9. Gender-based analysis for *GZMB, NRP1, PDCD1, FASLG, TNFRS18, SERPINB9* and *ITPR1* transcripts in GV Tregs and control Tregs.**

a) *GZMB* transcript levels in Tregs of male and female GV patients (p=0.2462). b) *NRP1* transcript levels in Tregs of male and female GV patients (p=0.1739). c) *PDCD1* transcript levels in Tregs of male and female GV patients (p=0.5642). d) *FASLG* transcript levels in Tregs of male and female GV patients (p=0.4852). e) *TNFRS18* transcript levels in Tregs of male and female GV patients (p=0.6737). f) *SERPINB9* transcript levels in Tregs of male and female GV patients (p=0.5742). g) *ITPR1* transcript levels in Tregs of male and female GV patients (p=0.4513).

**
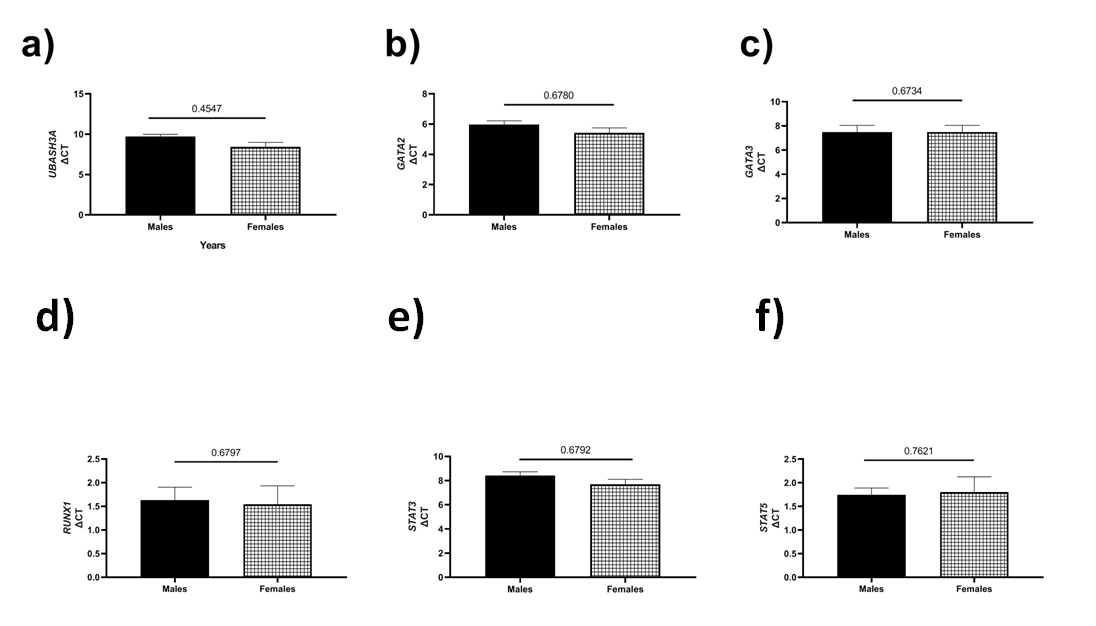
**

**Figure S10. Gender-based analysis for *UBASH3A, GATA2, GATA3, RUNX1, STAT3,* and *STAT5* transcripts in GV Tregs and control Tregs.**

a) *UBASH3A* transcript levels in Tregs of male and female GV patients (p=0.4547). b) *GATA2* transcript levels in Tregs of male and female GV patients (p=0.6780). c) *GATA3* transcript levels in Tregs of male and female GV patients (p=0.6734). d) *RUNX1* transcript levels in Tregs of male and female GV patients (p=0.6797). e) *STAT3* transcript levels in Tregs of male and female GV patients (p=0.6792). f) *STAT5* transcript levels in Tregs of male and female GV patients (p=0.7621).
